# Constitutive Gene Expression in Monocytes from Chronic HIV-1 Infection Overlaps with Acute Toll-Like Receptor Induced Monocyte Activation Profiles

**DOI:** 10.1371/journal.pone.0041153

**Published:** 2012-07-18

**Authors:** Bethsebah Gekonge, Malavika S. Giri, Andrew V. Kossenkov, Michael Nebozyhn, Malik Yousef, Karam Mounzer, Louise Showe, Luis J. Montaner

**Affiliations:** 1 The Wistar Institute, Philadelphia, Pennsylvania, United States of America; 2 Philadelphia Field Initiating Group for HIV-1 Trials, Philadelphia, Pennsylvania, United States of America; Wayne State University, United States of America

## Abstract

Elevated TLR expression/signalling in monocyte/macrophages has been shown to mediate systemic immune activation, a hallmark of progressive HIV-1 infection. Here we show, via differential gene expression comparisons, the presence of a constitutive *in vivo* TLR-like gene activation signature in steady-state circulating monocytes from chronically HIV-1 infected subjects. The TLR2-like gene signature was defined as an 82 gene subset of the 376 genes constitutively modulated in *in vivo* HIV-1 monocytes, based on their overlap with *de novo* TLR2-induced genes in uninfected subjects’ monocytes following acute *ex vivo* stimulation with *Staphylococcus Aureus Cowan* (SAC). Additional comparison of *in vivo* gene networks with available datasets from acute TLR activations in M/M expanded the overlap to 151-gene concordance among the 376 differential genes with emphasis on ERK/MAPK, TNF/IL6 (NFκB) and p53 gene networks. TLR2 stimulation of monocytes from HIV-1 infected subjects resulted in further upregulation of inflammatory genes indicative of a sustained transcriptional potential upon stimulation. In summary, our data support the presence of a sustained TLR-like gene activation profile in circulating monocyte from steady-state viremia in HIV-1 infected subjects.

## Introduction

Monocyte/macrophages (M/M) play an important role in HIV-1 infection. In addition to participating in the host anti-HIV-1 innate immune response [1], [2], [3], M/M are one of two primary cellular targets of HIV-1 [4]. M/M bind HIV-1, support virus replication [5], and serve as quiescent and long-lived viral reservoirs [6], [7], [8]. Several *in vitro* studies have shown functional and metabolic impairments in M/M following infection/exposure to HIV-1 [9], [10], [11], [12], [13]. Studies of global gene modulation *in vitro* in HIV-infected MDMs have identified HIV-induced macrophage activation profiles such as the modulation of pro-viral transcription factor genes, inflammatory, and modulation of cell cycle genes that have all been proposed to contribute to sustained viral replication [14], [15]. Unlike *in vitro* MDMs exposed to infectious HIV-1 where the primary contribution to gene modulation is from productive HIV-1 replication in MDM over time, the modulation of the *in vivo* circulating monocyte cell subset is not associated with productive monocyte infection as less than 1% are infected, gene modulation is expected to be the summation of HIV-1 virion-induced systemic changes and host-associated factors [16].

Toll Like Receptor (TLR)-mediated signalling has emerged as a major factor apart from HIV-induced signalling potentially contributing to the persistent chronic immune activation state observed during viremia. The notion that multiple TLR receptor activation outcomes could contribute to HIV-1 pathogenesis has been based on increased levels of circulating bacterial wall products in association with increased microbial translocation [17], the upregulation of TLR expression in immune cells including monocytes following HIV-1 infection [18], [19], the identification of HIV encoded TLR ligand interactions [20] and HIV-RNA mediated upregulation of TLR expression [21]. These observations question whether *in vivo* circulating monocytes in HIV-1 infection exhibit gene expression patterns similar to those elicited by specific TLRs [17]. To our knowledge, no direct comparison has been made in HIV-infected persons’ circulating monocyte gene expression *in vivo* with acute gene signatures for TLR-mediated gene activation programs in monocytes. Here, we have compared whether differential steady-state genes from circulating monocytes from HIV-infected subjects versus uninfected overlapped with *de novo* induced Toll-like receptor 2 gene signatures induced from uninfected subject monocytes.

## Materials and Methods

### Donors and Cell Subset Isolations

Chronically HIV-seropositive viremic patients, with a mean age of 43 years and not on therapy from the Jonathan Lax Immune Disorder Clinic (Philadelphia Field Initiation Group for HIV Trials) served as our donor population for microarray experiments. For inclusion, CD4 T cell counts were >200 cells per mm^3^ (mean of 460 cells/mm^3^), and viral load >10,000 copies/ml (mean of 32,000 copies/ml) (Table 1). HIV donors were selected if asymptomatic with no clinical evidence of active comorbidity (from hematocrit, body temperature, presentation/history). Age- and gender-matched healthy HIV-1-seronegative donors from the Wistar Institute Blood Donor Program were included as control subjects. Institutional Review Board approval (from the Wistar Institute and Philadelphia Field Initiation Group for HIV Trials) and informed consent were obtained before blood donation. As with HIV-infected donors, uninfected donors with an abnormal temperature, abnormal hematocrit, or reporting any symptoms were excluded. Blood was processed within 2–3 h from drawing. All reagents used were selected for their low levels of endotoxin contamination. Methods for monocyte isolation and purity assessment have been previously described [16]. Briefly, PBMC were separated by Ficoll Paque (Amersham Pharmacia Biotec) density gradient centrifugation, and monocytes were isolated by adherence enrichment for gene expression analysis or by negative selection following column purification for flowcytometry-based assessments for IL-1ß and TNF-α expression (Miltenyi Biotec). The mean purity of CD14^+^ monocyte preparations used in the study was 95% (±3%).

**Table 1:** HIV-1 Subject Characteristics.

Donor ID	Experiment	CD4 Count (cells/cubic mm)	Viral Load RNA (copies/ml)	Sex	Age(Years)
1	microarray	352	22,170	F	48
2	microarray	458	23,262	M	46
3	microarray	300	10,611	M	40
4	microarray	353	24,075	M	54
5	microarray	332	29,587	F	46
6	microarray	306	3,283	F	55
7	microarray	504	61,470	M	36
8	microarray	201	56,643	M	35
9	microarray	1128	9,810	F	35
10	microarray	416	74,296	M	38
11	microarray	896	18,088	F	39
12	microarray	604	22,150	F	20
13	microarray	168	61,432	M	33

1–13 served as donors for microarray experiments. Donors were asymptomatic and had a history of viremia with >10,000 copies of virus/ml of plasma and were not on therapy. Viral load and CD4 T cell count measured on the day of blood draw are indicated.

### RNA Isolation, Amplification, and Hybridization

Total cellular RNA was extracted from individual enriched monocytes of 13 viremic HIV-1 and 12 control uninfected donors within 20 hours of isolation using TRI reagent (Molecular Research Center, Inc., OH) from both unstimulated and 5-hour *Staphylococcus aureus* Cowan Strain (SAC) (0.2µg/ml) stimulated monocytes. Samples were isolated and analyzed individually consisting of 13 monocyte isolations from HIV infected subjects (P) and 13 paired 5h SAC stimulated (PS); 12 monocyte isolations from uninfected subjects as controls (C) and 12 paired 5h SAC Stimulated (CS). One microgram of total RNA was linearly amplified as previously described [22]. [*α*-^33^P] dCTP-labeled cDNA probes (1.6 *µ*g) were hybridized to cDNA arrays (manufactured at the Wistar Institute Microarray Facility) that contained 19,200 human probes representing ∼14,000 known genes. All amplifications and hybridizations were performed in a single batch to minimize experimental variations. Arrays were subjected to high stringency washes, exposed to PhosphorImager screens (Packard Instruments) for 7–10 days, scanned in a Storm 820 PhosphorImager, and visualized using ImageQuant (Molecular Dynamics) [22].

### Gene Expression Analysis and Pathway Analysis

Analysis of significant genes and of overrepresented functional groups, pathways and gene networks associated with significant and differentially expressed genes were carried out as previously described [16]. Statistically significant and differentially expressed genes between patient and control groups had a p≤0.05 and absolute fold change value (AFC) ≥2. The number of significant genes obtained was greater than the expected number of false positives. When identifying differentially expressed genes using quantile-normalized and log_2_-transformed data comparing 13 isolated monocyte samples from HIV viremic subjects (P) and paired SAC stimulated samples (PS); 12 uninfected monocyte controls (C) and paired SAC Stimulated samples (CS), FDR estimates [23] for p≤0.01 were 0.56 for P vs. C, 0.418 for PS vs. CS, 0.408 for C vs. CS and 0.26 for P vs. PS. We correlated differential gene expression with viral load data and used three different approaches to explore the functional relationships among the genes identified: DAVID (Database for Annotation, Visualization, and Integrated Discovery; david.abcc.ncifcrf.gov/), the Ingenuity Pathway Analysis tool (www.ingenuity.com), and Pathway Miner tool version 1.1 (BioRag, Bio Resource for Array Genes, at www.biorag.org).

### Correlation Heatmap

Pearson correlation between each pair of samples was calculated using expression for all 19,200 probes present for the microarray platform. Pearson correlation coefficient values were colour-coded to facilitate the generation of the correlation heatmap.

### Expression Heat Map

The list of 82 genes found to overlap between differential genes in HV infection and *de novo* TLR2-induced genes from uninfected subject monocytes was organized using a hierarchical clustering with Spearman correlation distance and complete linkage. A Heatmap showing expression of the 82 genes was plotted using colour intensity proportional to a value calculated as a ratio between gene expression in a sample and geometric mean of the gene across all samples.

### Principal Component Analysis

To visualize similarities between samples, we performed Principal Component Analysis using expression of the 82 genes and projected each sample on the 1^st^ and 2^nd^ principal components.

### Expression Profiles

We modelled expression in sample groups to define 6 expression profiles summarized by threshold fold changes relative to fold change differences observed relative to each group (C, CS, P, PS) respectively as follows: a) 1,2,2,2 (i.e.: C as reference group with 2 fold increases observed in CS, P, and PS); b) 2,1,1,1; c) 1,2,2,4; d) 4,2,2,1; e) 1,2,2,1; f) 2,1,1,2. Each of the 82 genes was assigned to one of those profiles, which showed the best Pearson correlation coefficient with the expression of the gene. Gene expression was averaged for every group of samples (C, CS, P, PS).

### Samples Clustering

Samples were clustered with hierarchical clustering using standardized Euclidean distance and complete linkage.

### Gene Expression Data

The microarray gene expression data discussed in this publication have been deposited in National Center for Biotechnology Information’s Gene Expression Omnibus (GEO) and are accessible through GEO Series accession no. GSE14542 (www.ncbi.nlm.nih.gov/geo).

### Flow Cytometry

For surface and intracellular protein measurements, PBMCs were obtained from healthy uninfected controls and viremic HIV-1 subjects by Ficoll Paque centrifugation. All antibodies were obtained from BD BioSciences and titrated to determine the appropriate saturating concentrations. These concentrations were in the range of 0.5–1 µg/million cells for all Abs used. Isotype-matched mAbs were used in each staining experiment to determine gates for positive events in monocyte subsets in all experiments. One million PBMCs were stained at 4°C for 30 minutes in the dark with surface antibodies against CD14 (catalog no. 555399) to identify monocytes and CD4 (catalog no. 555347). For intracellular protein expression in unstimulated or 5-hour SAC stimulated monocytes, anti-CD14 was used in fixed/permeabilized cells with intracellular antibodies against TNFα (catalog no. 555583) and IL1β (catalog no. 340518) using the protocol provided by BD Biosciences (BD Cytofix/Cytoperm Permeabilization Kit; catalog no. 554714). One hundred thousand events were acquired per stain from a live cell gate based on size and granularity on a FACS Caliber flow cytometer and analyzed using FlowJo software (Tree Star).

### Statistical Comparisons

For analysis of significance between unrelated groups, unpaired two-tailed Student’s t test were used if samples exhibited a normal distribution, a Wilcoxon two-sample test was used when samples exhibited non-normal distribution, and a two-tailed paired Student’s t test was used to compare paired treatment groups. For all tests, a two-sided p value ≤0.05 was considered significant. All tests were performed using JMP (SAS Institute) statistical software.

## Results

### Similarity between Steady-state Gene Expression Signature in Monocytes from *in vivo* HIV-1 Infection and following *in vitro* TLR Stimulation

**Figure 1:**
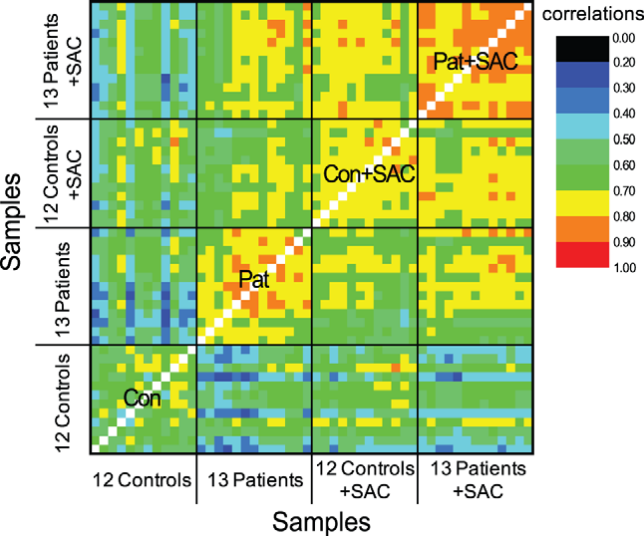
Higher correlation between HIV-1 samples at steady state and following TLR2 stimulation with SAC. The correlation heatmap shows sample to sample correlation between the 13 HIV-1 (P), 12 control (C), 13 SAC stimulated HIV-1 (P+SAC) and 12 SAC stimulated control (C+SAC) samples based on expression of all genes included in array analysis and following exclusion of background (noisy) genes. HIV-1 samples at steady state and following TLR2 stimulation with SAC (P and P+SAC) exhibit greater within group correlation on the basis of monocyte gene expression. The order of samples along the two axes is: controls – 1–12, patients - 1–13, controls stimulated with SAC - 1–12 and patients stimulated with SAC −1–13. Correlation among samples within each group is indicated for the four groups with labels Con, Pat, Con+SAC and Pat+SAC. The legend indicates correlation values for the different colors.

To investigate the functional consequences of acute *ex vivo* stimulation of HIV-1 monocytes on constitutive gene expression, we compared circulating monocytes from 12 uninfected versus 13 HIV-1 viremic subjects after a 5-hour exposure to *Staphylococcus aureus Cowan* (SAC) that elicits signalling through the monocyte TLR2 receptor [24], [25]. We selected TLR2 as the activation stimulus since we have previously characterized TLR2 signalling defects in PBMCs isolated from HIV-infected subjects and because increased soluble TLR2 levels, as well as enhanced TLR2 expression in circulating monocytes in HIV-infected subjects, has been previously reported [18], [19]. Interestingly, correlation analysis taking into account all genes within the four study groups, clearly demonstrated a greater sample-to-sample correlation in the HIV-1 viremic (P) samples when compared to samples within the control group (C) before TLR stimulation (Fig. 1). The impact of TLR stimulation further increased similarities in both uninfected or HIV-infected monocytes as evidenced by correlation heat map analysis. These results support a greater homogeneity of gene expression in HIV viremia within circulating monocytes than what is found in uninfected persons. Second, a higher sample-to-sample correlation was further achieved following TLR2 stimulation in both HIV viremic and uninfected monocytes (Fig. 1). Results are consistent with a coordinated gene expression following SAC stimulation consistent with uniform stimulation as provided *ex vivo*.

### Overlap between steady-state gene expression signature in monocytes from *in vivo* HIV-1 infection and uninfected monocyte gene expression following *in vitro* TLR stimulation

**Figure 2:**
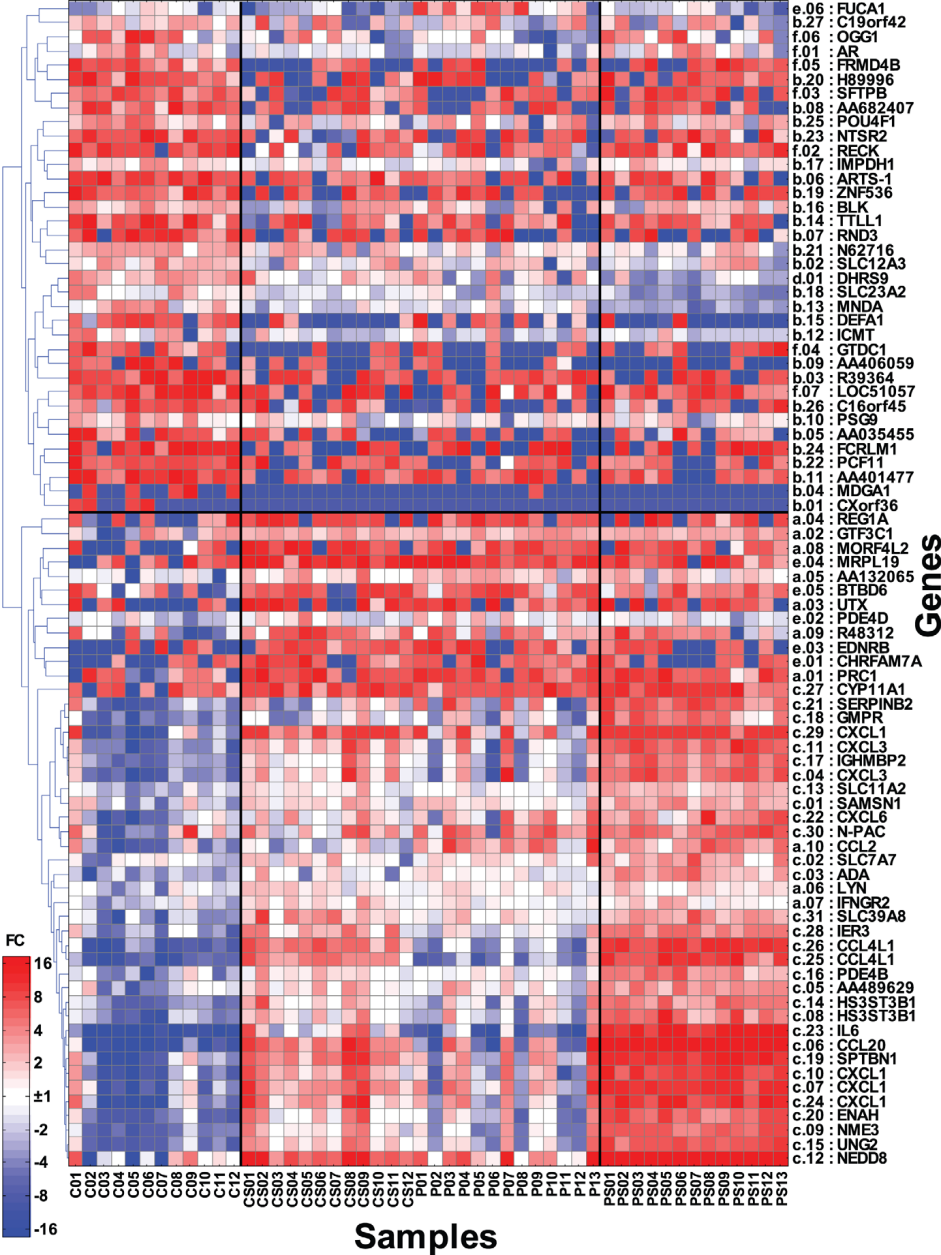
82 genes with TLR2 gene profile in steady-state HIV-1 monocytes in vivo. Heatmap of expression values for 82 genes across 13 HIV-1 (P), 12 control (C), 13 SAC stimulated HIV-1 (PS) and 12 SAC stimulated control (CS) samples. Genes are clustered using spearman correlation distance. Each gene is represented by its symbol, assigned expression profile (a – f) and index as indicated on Figure 3C and also in gene listing in Table S2.

**Figure 3:**
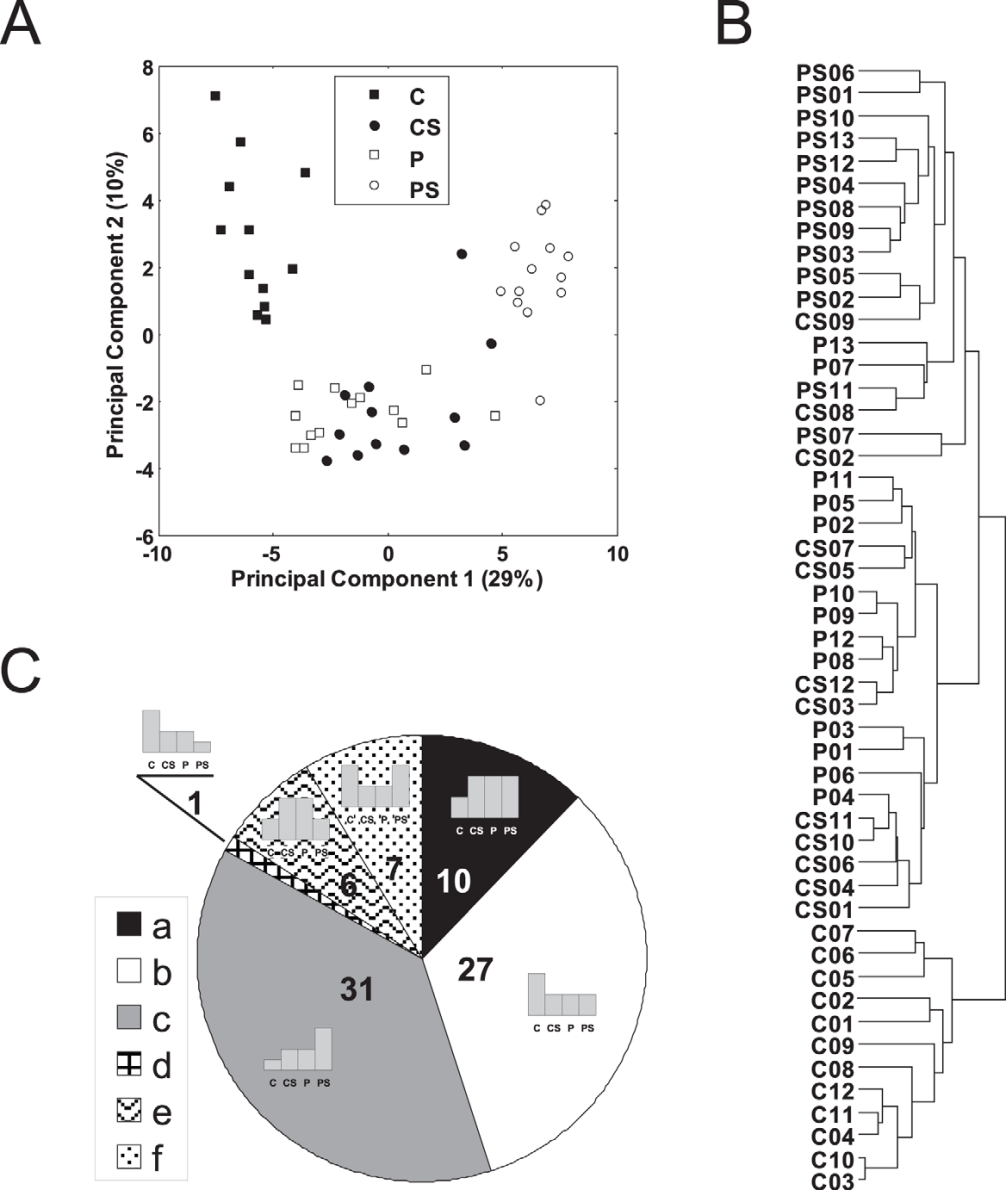
Principal component analysis, hierarchical clustering and expression pattern clusters for all groups analyzed. (A) Similarities between samples based on Principal Component Analysis. Expression of 82 genes listed in Figure 2 was used to project each sample on the 1^st^ and the 2^nd^ principal component. (B) Hierarchical clustering of samples. Standardized Euclidean distance between samples calculated based on expression of 82 genes was used. (C) Expression variability according to six predefined expression profiles (a – f; see Table S2) as described in methods. Each of the 82 genes was assigned to only one expression profile that showed the best correlation with the gene expression. Group assignment between a-f is also listed per individual gene in Figure 2 and Table S2.

To further characterize the TLR gene profile, we compared induced genes in uninfected SAC-stimulated monocytes (i.e., 458 genes) against the constitutive steady-state gene expression in HIV viremic monocytes (i.e., 376 genes), and identified an overlap of 82 genes (Listed in Table S2 and listed in Fig. 2). Expression levels for these genes were not correlated with respective HIV donor plasma viral load. In order to study the relationship between the total 50 samples analyzed in regards to their relative expression of these 82 genes, we used Principal Component Analysis and visualized relationships using the first two principal components (Fig. 3A) and also performed hierarchical clustering on those samples (Fig. 3B). As seen in both Figure 3A and Figure 3B, samples from the Control group and the activated Patient group formed three separate clusters. The first cluster showed all control unstimulated samples, the second cluster showed stimulated control samples together with unstimulated HIV viremic samples and the third cluster showed stimulated HIV viremic samples. Both analysis approaches demonstrate that modulation of gene expression by SAC-stimulation in normal monocytes is similar to steady-state gene signatures in monocytes in HIV-1 infection.

To characterize the 82 overlap genes and identify the specific genes that are driving these cluster patterns between the four classes of samples (C, CS, P, PS), we defined 6 basic expression profiles for each of the 82 genes reflecting the relationship between the 4 groups of samples (Fig. 3C). Table S2 includes the name of each gene included in the 6 expression profiles shown by usage of letters a-f in conjunction with listing ID# 1–82. Please note that the same gene expression pattern id shown here with letter a-f is also listed on Figure 2 to show individual genes within each expression pattern. The biggest group represented by 37 genes (45%), comprised expression profiles “a” and “b” as defined in the methods section and listed on Figure 2, includes genes that are TLR2 induced in control samples yet already regulated to the same degree (up- or down-regulated) in viremic subjects without change following exogenous TLR2 stimulation. In contrast, profiles “c” and “d” (32 genes) comprised genes that were modulated by TLR2 stimulation in viremic samples representing the genes that were further up- or down- regulated following stimulation. This profile included inflammation associated genes such as *CXCL1, CXCL3, CCL20*, *SERPINB2/PAI2, CCL4L1,* and *IL-6*, consistent with ongoing inflammatory gene expression. The remaining two patterns (“e” and “f”) show genes that like patterns “a” and “b” are already regulated in viremic subjects as found after induction of TLR2 in control samples yet their pattern of expression changes after TLR2 stimulation in viremic subjects.

### Over-expressed Constitutive TLR-like Overlap Genes in HIV Subject Monocytes are not Refractory to further Stimulation after TLR-2 Stimulation

**Figure 4:**
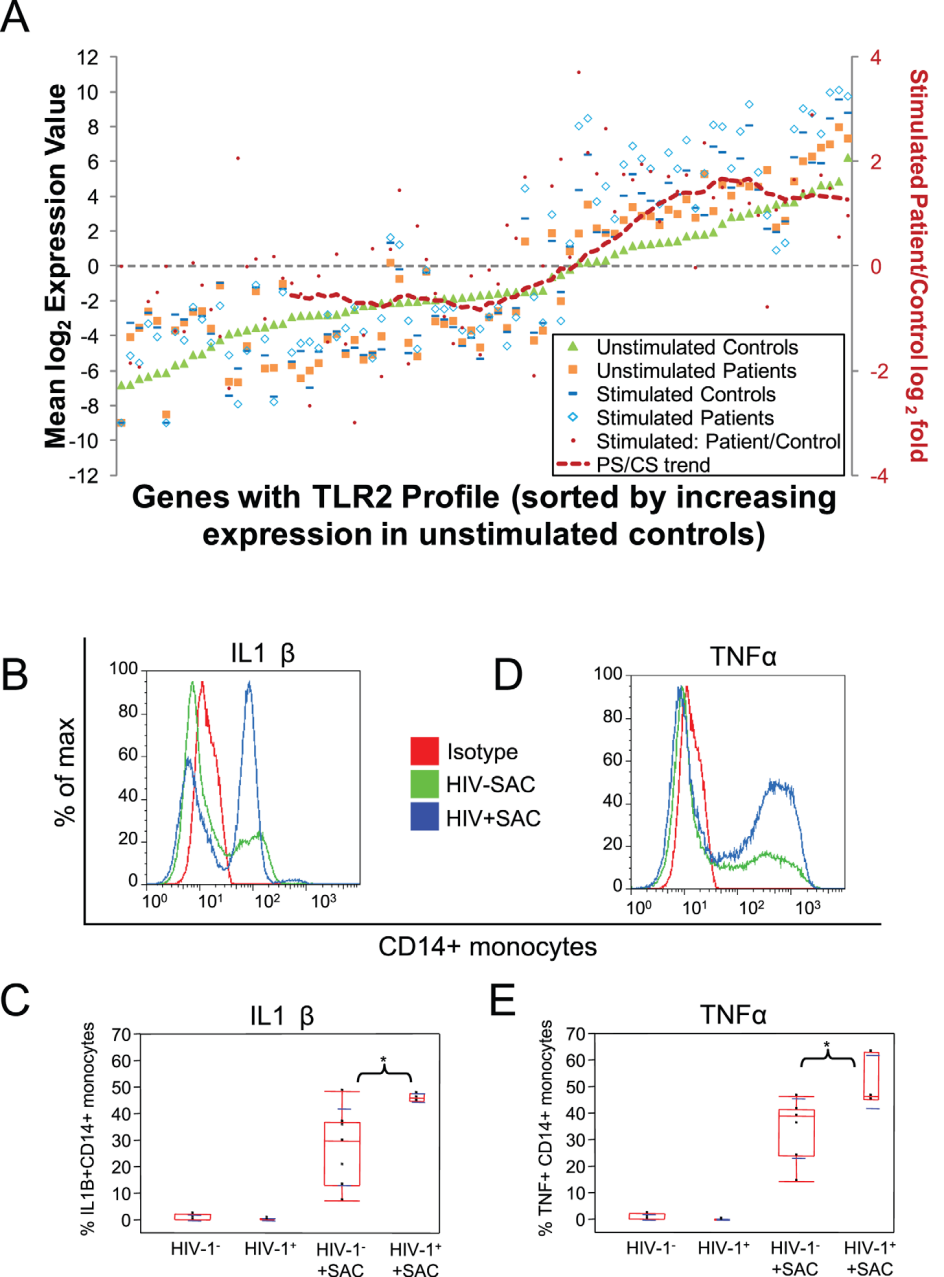
Increased gene dysregulation of the 82 TLR2 gene profile by *ex vivo* SAC (TLR2) stimulation. (A) The 82 genes are shown along the X-axis and mean log_2_ expression values in 12 controls (C, green), 13 HIV-1 donors (P, orange) and 12 SAC stimulated controls (CS, dark blue) and 13 SAC stimulated HIV-1 donors (PS, light blue) are shown along the Y-axis. Genes are sorted by increasing expression in the unstimulated control samples. Red dots indicate changes in PS gene expression above (positive fold change) or below (negative fold change) CS along the Y-axis on the right. Trend line is a moving average for the red dots with a window of 20 genes that demonstrates genes with higher expression (reason why the red line starts after 20 genes). Raw mean log_2_ expression values for the 82 genes for 12C, 13P, 12CS and 13PS are in Table S1. 56% (46/82) of the genes that comprise the constitutive TLR2 like activation signature of modulated HIV-1 monocyte genes exhibit enhanced dysregulation following acute *ex vivo* SAC (TLR2) stimulation. Histograms in (B) and (D) show enhanced expression of IL1β and TNFα protein in HIV-1 than in control CD14 monocytes following *ex vivo* stimulation with SAC (via TLR2) for five hours. Summary statistics represent median +/− s.d. for percent CD14 monocytes IL1β (C) and TNFα (E) in 7 control donors and 3 HIV-1 donors. “*” represents significance (p≤0.05).

We analysed the extent of change in the target 82 shared constitutive activation genes following TLR2 stimulation of monocytes from HIV-infected subjects (Fig. 4A). It is interesting to note that stimulation of patient samples results in enhanced dysregulation, approximately 4 fold (log_2_ = 2) more than the effect of stimulating control samples in 56% (46 of 82) of target genes (Fig. 4A). Overall, we observed an amplification of the constitutive expression level of pro-inflammatory cytokines, chemokines and growth factors (*TNF, CD40, CCL18, IL1A, IL1B, CSF2, CSF3*), transcription factors (*STAT5A, STAT4, NFκB*) and inhibitors of signalling and transcription (*SOCS3, CIS*). Gene expression data after stimulation confirmed that differential steady-state gene expression associated with inflammation was not refractory to further stimulation and an increase in coordinated gene expression as already suggested by Figure 1. As illustrated already by pattern “c” and “d”, supporting the potential for HIV viremia to facilitate a greater inflammatory response upon TLR2 stimulation, we observed higher intracellular protein levels of inflammatory cytokines IL1β and TNF in HIV-1 vs. control monocytes following acute *ex vivo* SAC stimulation (Fig. 4, B-E).

### Concordance between Functional Gene Modulation *in vivo* Monocytes and *in vitro* Stimulated Monocytes

As we had previously identified 46 genes that were overlapping between the 376 *in vivo* differential genes when compared against three available datasets (Table 2) in which MDM exhibited differential gene expression following acute exposure via HIV-1 gp120 and/or infection with HIV-1 [26], [27], [28], we determined if any of the target overlap 82 TLR induced-like genes were also present within these *in vitro* HIV-stimulated genes. We found that 13 of the 82 genes (16%) described here overlapped, suggesting that the majority of the constitutive TLR2-like gene expression in HIV viremic monocytes is not associated with direct HIV interactions as modelled *in vitro* and likely represents the presence of additional factors such as microbial translocation [17] and/or host factors. To further evidence the potential contribution of microbial translocation-related factors *in vivo* and recognising that TLR2 may represent but one of several TLR-mediated activation outcomes, we took advantage of the availability of four independent gene expression studies examining acute *in vitro* TLR-mediated stimulations of M/M and further evidenced overlap of constitutively activated genes from 82 to 151 within the 376 differentially expressed genes in circulating HIV monocytes at steady-state (Tables S1 & Table 3). While this additional analysis supports the presence of overlapping TLR-induced genes within circulating monocytes, the different approaches for defining the magnitude of expression between studies did not allow for direct quantitative comparisons. Further studies will need to expand on direct comparisons between constitutive gene expression within circulating monocytes in HIV-1 pathogenesis and matched TLR stimulations in monocytes from uninfected persons beyond TLR2 as we interpret the 82 genes identified here may represent a subset of the full TLR-like signature sustained *in vivo*.

**Table 2:** Description of HIV-1 M/M Datasets Used For Comparative Analyses.

Dataset ID	Study Title	PMID	Array Platform	Differential Genes Reported
Cicala C	Acute exposure of MDM to HIV-1 JRFL gp120	[26]	Affymetrix Human Genome U95A oligonucleotide arrays	939 genes investigator provided
Coberley CR	One week MDM infection with HIV-1 JRFL	[27]	Affymetrix Human Genome U95A oligonucleotide arrays	821 genes investigator provided
Vazquez N	One week MDM infection with HIV-1 BAL	[28]	Atlas Human cDNA expression array (1.2 I; Clontech)	364 genes investigator provided

Indicated are the study ID, study description, reference, microarray platform used and number of differential genes reported for HIV-1 monocyte/macrophage (M/M) gene datasets used for comparison against our *in vivo* gene lists.

**Table 3:** Description of non HIV-1 M/M Datasets Used For Comparative Analyses.

Dataset ID	Agonist	Study Title	PMID	Array Platform	Differential Genes Reported
GSE3982	LPS	Human immune cell transcriptome	[33]	Affymetrix GeneChip Human Genome U133 Array Set HG-U133A	Do not report a
GSE2135	Filarial worms	Filaria induced monocyte dysfunction and its reversal following treatment	[34]	Affymetrix GeneChip Human Genome U133 Array Set HG-U133A	62
GDS260	Bacteria	Pathogen exposure and immune response	[35]	Affymetrix GeneChip Human Genome U95 Set HG-U95A	1060
Nau GJ	Bacteria and Bacteria components	Human macrophage activation programs induced by bacterial pathogens.	[36]	Affymetrix Hu6800 GeneChips	977

Indicated are the study ID, agonist, study description, reference, microarray platform used and number of differential genes reported if available for TLR monocyte/macrophage (M/M) gene datasets used for comparison against our *in vivo* gene lists.

a indicates differential genes were calculated by using analysis approach described in methods.

### Gene Network Analysis of Overlap TLR-like Induced Genes in HIV Subject Monocytes Identifies Constitutive Clusters for TNF (NFκB), p53 and MAPK Networks

**Figure 5:**
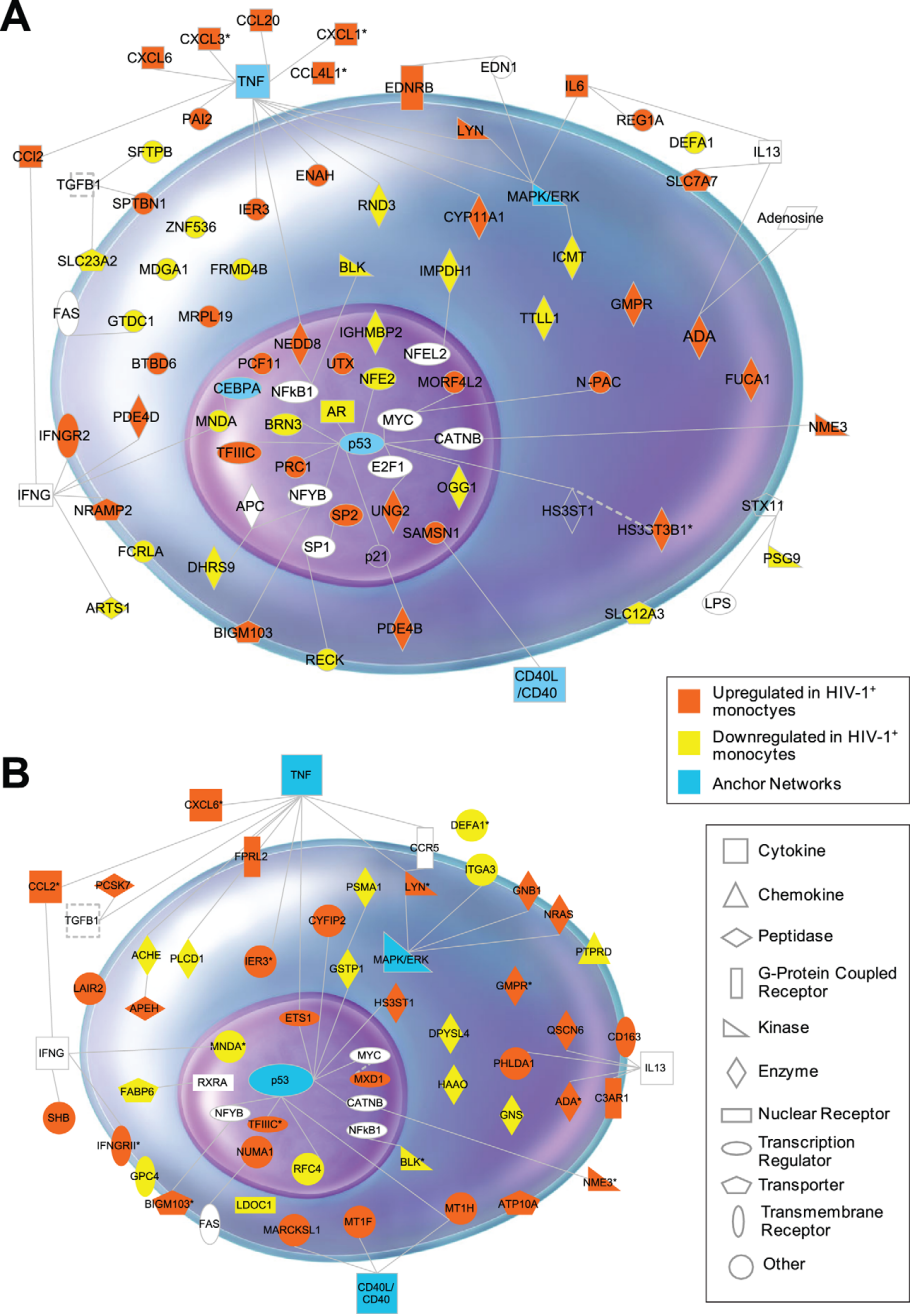
TLR2 and HIV-1 associated gene signatures in HIV-1 monocytes in vivo. (A) 22% (82 of 376) of genes constitutively expressed in circulating monocytes from HIV subjects were defined as TLR2 specific as they were overlapping with TLR2 mediated gene regulation in control monocytes. The cellular location and function of 63 genes of this 82 TLR2 profile was mapped by network analysis (B) 12% (46 of 376) of genes were defined as HIV-specific as they were constitutively expressed in circulating monocytes from HIV subjects and overlapping with HIV-1-mediated gene regulation in *in vitro* MDM. The 13 of the 46 genes overlapping with the TLR2 gene group shown in A are marked with an asterix. The accompanying legend defines the shapes used to represent the genes and the direction of gene expression in HIV-1 monocytes.

To define HIV-specific vs. disease (TLR)-specific gene regulatory effects, we performed gene pathway and network analyses using Ingenuity, Pathway Miner and DAVID as previously described [16]. We were able to map the cellular location and function for 63 of the 82 genes of the TLR2 profile (Fig. 5A). The majority of these genes were associated with inflammation and immune response functions and clustered into TNF (NFκB), p53 and MAPK networks. The TLR2 induced gene group included *PAI2, IER3, CCL2, CCL20, CXCL1, CXCL6, CXCL3, ADA, NEDD8, UTX, IGHMBP2, ENAH, IL6, ARTS1, CYP11A1, IFNGR2, SAMSN1* and *LYN,* all consistent with the known outcomes of acute TLR-induced gene expression [29] (Fig. 5A). Similar mapping of the 46 of 376 genes that overlapped with *in vitro* HIV-induced gene changes are shown in Figure 5B where the shared 13 genes with TLR2-induced changes are highlighted within same major networks.

## Discussion

Our results provide the first evidence for a TLR-like gene signature in circulating HIV-1 monocytes in the presence of chronic viral replication *in vivo.* Furthermore, we now provide added evidence in support of HIV pathogenesis resulting in a steady-state “mature” or activation-induced differentiation within circulating monocytes through both viral and circulating TLR ligands present during chronic viral replication [17], [30]. In this study, we directly compared the recently described 376 significant genes that exhibited constitutive differential modulation between unstimulated circulating monocytes from HIV-infected persons versus control monocytes [16] (Table S1) to determine (a) if similarities were present between steady-state gene signatures in monocytes in HIV-1 infection *in vivo* versus the *de novo* expression in monocytes from uninfected subjects induced by a TLR2 agonist, and (b) if constitutive gene expression in circulating monocytes from HIV infected persons was biased toward or against *de novo* gene modulation of activation genes following *ex vivo* stimulation via TLR2. We define for the first time an overlap between TLR-mediated activation of gene expression in monocytes with the constitutive gene expression of monocytes from HIV-infected subjects *in vivo*. Interestingly, HIV viremic monocytes already showed greater homogeneity in gene expression before stimulation, which was further increased after stimulation, consistent with a differential constitutive gene expression and retained ability to respond to TLR2 activation as also observed in stimulated control samples. TLR2 stimulation through SAC (S) induced differential gene expression within intra group comparisons (p≤0.05 and AFC ≥2) of HIV subject (P) or control subject (C) groups (“C vs. CS”; gene number - 458 and “P vs. PS”; gene number - 723 respectively) as well as showing differential inter-group gene expression if comparing induced genes (i.e., “PS vs. CS”; gene number - 475).

With regards to constitutive gene expression in circulating monocytes during viral replication in chronic infection, our study now expands on previous published reports by characterizing predominant gene networks that are similar to those elicited by inflammatory responses following *ex vivo* TLR2 stimulation [18]. Furthermore, our identified target overlap genes are interpreted when compared to other gene expression reports showing HIV-induced upregulation of transcriptional machinery required for inflammatory gene/protein expression (including TNF and IL1β) by macrophages [11], [26], [31] while corroborating that increases in classical pro-inflammatory cytokines may represent an enhanced response of already over-expressed proteins in on-going HIV-1 disease. Indeed, while we confirm an altered monocyte activation phenotype *ex vivo*, we also demonstrate that the functional activation by TLR *in vitro* further regulates already differentially modulated genes to indicate that inflammation in HIV-1 infection remains able to further upregulate gene expression. The latter is consistent with the “additive effect” on inflammatory gene expression described when comparing HIV infection versus co-infection with a bacterial pathogen where added TLR activation is proposed to further upregulate immune activation genes. Our gene expression data in monocytes from HIV subjects is consistent with a retained responsiveness to TLR activation (i.e., not refractory or tolerant) in spite of viral replication *in vivo*. It remains to be determined whether down modulation of virus inhibitor genes (e.g. ANXA1, SOCS3, CIS) will contribute to this over expression potential upon further TLR-mediated activation. Considering that circulating monocytes remain preferentially uninfected (i.e., <1% are identified as infected, [6]) and the lack of a correlation between the TLR-like induced constitutive gene expression and HIV subject viral load, our data supports the interpretation that gene expression in circulating monocytes among asymptomatic persons may be due to external conditioning (i.e., microbial translocation levels) and not as a result of direct monocyte HIV infection rate or fluctuations in viral load [32]. It is important however, to note that our data addresses mid-stage chronic viremia, therefore future studies will need to address the impact on monocyte gene expression of acute infection, end-stage disease or presence of co-morbidities with regards to our data. Furthermore, future investigation will need to determine if viral suppression following therapy, in association with a reduction of bacterial translocation and viral load, will also impact a reversal in the TLR-like gene signature observed in circulating monocytes as predicted by our data.

Our study supports the interpretation that circulating monocytes in HIV-1 infection are not “undifferentiated” or “resting” cells but represent a constitutively activated/differentiated or “mature” MDM-like monocyte with active differential gene expression likely to impact inflammatory monocyte function upon recruitment towards tissue macrophages and/or precursors to dendritic cells. While the presence of regulated gene expression in monocytes exposed to HIV-1 infection or viral products *in vitro* has long been documented, our data suggest that the described alterations in gene expression *in vitro* to date may be a small part of the active gene expression networks present *in vivo*.

Taken together, our observations (i) provide clear evidence for steady-state activation in circulating monocytes with the potential for amplification of this response with additional stimulation, (ii) show distinct overlaps between *in vivo* (constitutive) and *in vitro* elicited gene expression changes from both virion-mediated and TLR-mediated changes, and (iii) suggest a benefit in targeting the analysis of monocyte gene expression as a potential marker to track gene patterns that may reflect distinct microbial translocation or virion-induced changes which may bear on defining stages of disease progression in HIV-infected subjects as well as monocyte regulatory changes during active co-morbidities.

## Supporting Information

Table S1 **376 Constitutively modulated genes in HIV-1 monocytes in vivo.** Spot IDs, accessions, gene symbols, gene names, p values and ratios for 376 significantly and differentially modulated genes between 13 HIV-1 and 12 control donor monocytes are shown. Also shown are p values and ratios for the 82 genes within the 376 P vs. C genes that are significantly and differentially modulated by SAC in control donor monocytes (C vs. CS) and constitute the TLR2 profile. The average raw Log_2_ values for these 82 genes that were used for generation of the plot in Figure 4B for 12C, 13P, 12CS and 13PS are also provided. p values and ratios for group comparison PS vs. CS for 92 of 376 constitutive genes that continued to maintain differential expression following SAC stimulation in HIV-1 monocytes are shown. Genes shared with 3 *in vitro* HIV-1 databases are indicated (HIV-1) as are genes shared with 7 additional databases obtained from non-HIV-1 TLR stimulations of monocytes (other). The *in vivo* genes cumulatively accounted for by HIV-1 and non-HIV-1 databases are shown (accounted) and the remaining as yet unaccounted genes are shown (unaccounted). Genes that indicated the same expression trend in the 7 month resampled and rehybridized group of 4 HIV-1 and 4 control donors are additionally shown. * indicates multiple accessions (multiple clones) for the gene. Genes upregulated in P, CS or PS in comparison to C, C, and CS respectively have a positive value and genes downregulated have a negative value. p values <0.005 are indicated as 0.00.
(DOC)

Table S2 **Summary of fold changes in the 82 genes between average expression values for 4 different groups of samples.** *r*  =  Pearson correlation between expression of a gene and the assigned expression profile (a-f) as “ID” from grouping shown in Figure 3C and also listed on Figure 2. pval  =  minimal p-value for the gene.
(DOC)
