# Supplementary material for: Constitutive Gene Expression in Monocytes from Chronic HIV-1 Infection Overlaps with Acute Toll-Like Receptor Induced Monocyte Activation Profiles
Source: PLoS One. 2012 Jul 18;7(7):e41153. doi: 10.1371/journal.pone.0041153 (PMC3399809; doi:10.1371/journal.pone.0041153)
Supplement: Table S1 — 376 Constitutively modulated genes in HIV-1 monocytes in vivo. Spot IDs, accessions, gene symbols, gene names, p values and ratios for 376 significantly and differentially modulated genes between 13 HIV-1 and 12 control donor monocytes are shown. Also shown are p values and ratios for the 82 genes within the 376 P vs. C genes that are significantly and differentially modulated by SAC in control donor monocytes (C vs. CS) and constitute the TLR2 profile. The average raw Log2 values for these 82 genes that were used for generation of the plot in Figure 4B for 12C, 13P, 12CS and 13PS are also provided. p values and ratios for group comparison PS vs. CS for 92 of 376 constitutive genes that continued to maintain differential expression following SAC stimulation in HIV-1 monocytes are shown. Genes shared with 3 in vitro HIV-1 databases are indicated (HIV-1) as are genes shared with 7 additional databases obtained from non-HIV-1 TLR stimulations of monocytes (other). The in vivo genes cumulatively accounted for by HIV-1 and non-HIV-1 databases are shown (accounted) and the remaining as yet unaccounted genes are shown (unaccounted). Genes that indicated the same expression trend in the 7 month resampled and rehybridized group of 4 HIV-1 and 4 control donors are additionally shown. * indicates multiple accessions (multiple clones) for the gene. Genes upregulated in P, CS or PS in comparison to C, C, and CS respectively have a positive value and genes downregulated have a negative value. p values <0.005 are indicated as 0.00. (DOC) [file pone.0041153.s001.doc]

Table SI

| **Table SI: 376 constitutively modulated genes in HIV-1 monocytes *in vivo*** | | | | | | | | | | | | | | | | |
| --- | --- | --- | --- | --- | --- | --- | --- | --- | --- | --- | --- | --- | --- | --- | --- | --- |
|  |  |  |  |  | |  | | **Steady-state**  **TLR2 –like signature** | | **TLR2 82 gene profile**  **Raw mean Log_2_ values** | | | | ***in vitro***  **HIV-1/ MDM** | **Other**  **DB** | **Total**  **Accounted** |
| **Spot Id** | **Genbank Accession** | **Gene Name** | **Gene Symbol** | **Unstimulated**  **Patient/ Control** | | **Stimulated**  **Patient/ Control** | | **Control**  **Stimulated/**  **Unstimulated** | | **Control** | | **Patient** | |  |  |  |
|  |  |  |  | ***P*** | **Fold** | ***P*** | **Fold** | ***P*** | **Fold** | **Unstim** | **Stim** | **Unstim** | **Stim** |  |  |  |
| **10** | AA775872 | Glypican 3 | GPC3 | 0.04 | -3.18 |  |  |  |  |  |  |  |  |  |  | No |
| **18** | W92812 | Pro-platelet basic protein (chemokine (C-X-C motif) ligand 7) | PPBP* | 0.01 | 3.11 | 0.02 | 2.19 |  |  |  |  |  |  |  | Yes | Yes |
| **45** | H90287 | Ubiquitously transcribed tetratricopeptide repeat, chrX | UTX | 0.03 | 7.73 |  |  | 0.04 | 5.89 | -6.12 | -3.51 | -3.12 | -4.05 |  | Yes | Yes |
| **85** | R55490 | Phospholipase C, delta 1 | PLCD1* | 0.04 | -5.17 |  |  |  |  |  |  |  |  | YES | Yes | Yes |
| **127** | AA521243 | Mitochondrial ribosomal protein L19 | MRPL19 | 0 | 8.72 |  |  | 0.01 | 9.48 | -4.21 | -0.95 | -1.09 | -2.26 |  | Yes | Yes |
| **155** | W46577 | Endothelial cell-specific molecule 1 | ESM1 | 0.03 | -4.08 |  |  |  |  |  |  |  |  |  | Yes | Yes |
| **272** | W74337 | Ubiquitination factor E4B (UFD2 homolog, yeast) | UBE4B | 0.04 | -5.07 |  |  |  |  |  |  |  |  |  | Yes | Yes |
| **406** | AI150762 | Psoriasis susceptibility 1 candidate 2 | PSORS1C2 | 0.01 | -7.44 |  |  |  |  |  |  |  |  |  |  | No |
| **430** | AA935273 | Chemokine (C-X-C motif) ligand 3 | CXCL3* | 0.02 | 3.66 | 0 | 2.71 | 0 | 11.57 | 2.87 | 6.19 | 4.78 | 7.74 |  |  | Yes |
| **440** | AA973928 | Immunoglobulin mu binding protein 2 | IGHMBP2 | 0.05 | 2.24 | 0 | 3.16 | 0 | 5.84 | 3.69 | 6.26 | 4.87 | 7.88 |  | Yes | Yes |
| **501** | AA464152 | Quiescin Q6 | QSCN6 | 0.01 | 2.06 | 0.01 | 1.71 |  |  |  |  |  |  | YES | Yes | Yes |
| **546** | H28710 | Endothelin receptor type B | EDNRB* | 0.04 | -5.93 |  |  |  |  |  |  |  |  |  | Yes | Yes |
| **568** | N58136 | Hypothetical protein LOC283755 | LOC283755 | 0.01 | -5.61 |  |  |  |  |  |  |  |  | YES |  | Yes |
| **604** | AA454668 | Prostaglandin-endoperoxide synthase 1 (prostaglandin G/H synthase and cyclooxygenase) | PTGS1* | 0.02 | 2.03 |  |  |  |  |  |  |  |  |  | Yes | Yes |
| **616** | T97181 | Platelet factor 4 (chemokine (C-X-C motif) ligand 4) | PF4 | 0.04 | 2.84 | 0.03 | 1.57 |  |  |  |  |  |  |  | Yes | Yes |
| **653** | W93369 | CHRNA7 (cholinergic receptor, nicotinic, alpha 7, exons 5-10) and FAM7A | CHRFAM7A/ CHRNA7 | 0.02 | 7.49 |  |  | 0.05 | 7.28 | -6.43 | -3.54 | -3.52 | -5.54 |  |  | Yes |
| **654** | AA495802 | Activity-dependent neuroprotector | ADNP | 0 | 2.54 |  |  |  |  |  |  |  |  |  | Yes | Yes |
| **664** | R40244 | Protein tyrosine phosphatase, receptor type, D | PTPRD | 0.01 | -7.56 |  |  |  |  |  |  |  |  | YES |  | Yes |
| **768** | T80846 | 3-hydroxyanthranilate 3,4-dioxygenase | HAAO | 0.02 | -5.3 |  |  |  |  |  |  |  |  | YES | Yes | Yes |
| **771** | H07878 | G protein-coupled receptor 19 | GPR19 | 0.01 | 9.82 |  |  |  |  |  |  |  |  |  |  | No |
| **776** | R60723 | Purinergic receptor P2X, ligand-gated ion channel, 4 | P2RX4 | 0.05 | 2.93 |  |  |  |  |  |  |  |  |  | Yes | Yes |
| **786** | H58953 | Nuclear factor (erythroid-derived 2), 45kDa | NFE2 | 0 | -8.69 |  |  |  |  |  |  |  |  |  | Yes | Yes |
| **814** | AA045436 | V-maf musculoaponeurotic fibrosarcoma oncogene homolog G (avian) | MAFG | 0.03 | -5.1 |  |  |  |  |  |  |  |  |  |  | No |
| **822** | AA487215 | Myosin, light polypeptide kinase | MYLK | 0.03 | -6.72 |  |  |  |  |  |  |  |  |  | Yes | Yes |
| **888** | AA455632 | RNA binding motif protein 15B | RBM15B | 0.03 | -6.46 |  |  |  |  |  |  |  |  |  |  | No |
| **930** | AA702114 | Type 1 tumor necrosis factor receptor shedding aminopeptidase regulator | ARTS-1 | 0.02 | -4.98 |  |  | 0.04 | -3.81 | -1.38 | -3.25 | -3.69 | -3.24 |  | Yes | Yes |
| **951** | AA464582 | Zinc ribbon domain containing1 | ZNRD1 | 0 | 13.73 |  |  |  |  |  |  |  |  |  |  | No |
| **1,001** | R94661 | Major histocompatibility complex, class I, E | HLA-E | 0.01 | 2.08 | 0 | 2.37 |  |  |  |  |  |  |  | Yes | Yes |
| **1,215** | H38839 | Interphotoreceptor matrix proteoglycan 1 | IMPG1 | 0.03 | -7.05 |  |  |  |  |  |  |  |  |  | Yes | Yes |
| **1,264** | AA424695 | Integrin, alpha 3 (antigen CD49C, alpha 3 subunit of VLA-3 receptor) | ITGA3 | 0.02 | -5.78 |  |  |  |  |  |  |  |  | YES | Yes | Yes |
| **1,285** | AA757754 | Dihydropyrimidinase-like 4 | DPYSL4 | 0.03 | -7.51 |  |  |  |  |  |  |  |  | YES | Yes | Yes |
| **1,296** | AA700832 | Retinol binding protein 1, cellular | RBP1 | 0.03 | 3.31 |  |  |  |  |  |  |  |  |  | Yes | Yes |
| **1,298** | AC013459 | Nuclear receptor coactivator 1 | NCOA1 | 0.03 | 5.43 |  |  |  |  |  |  |  |  |  | Yes | Yes |
| **1,312** | R89567 | Group-specific component (vitamin D binding protein) | GC | 0.01 | -6.4 |  |  |  |  |  |  |  |  |  |  | No |
| **1,314** | AA482119 | Inhibitor of DNA binding 3, dominant negative helix-loop-helix protein | ID3 | 0.03 | 2.65 |  |  |  |  |  |  |  |  |  |  | No |
| **1,341** | BC018761 | Immunoglobulin kappa constant | IGKC | 0 | 2.75 | 0 | 3.62 |  |  |  |  |  |  |  | Yes | Yes |
| **1,352** | AA424743 | MRNA; cDNA DKFZp686O10247 (from clone DKFZp686O10247) | N/ A | 0 | 2.31 |  |  |  |  |  |  |  |  |  |  | No |
| **1,386** | AA436564 | C-mer proto-oncogene tyrosine kinase | MERTK | 0 | 2.79 |  |  |  |  |  |  |  |  |  | Yes | Yes |
| **1,449** | AA975010 | H2.0-like homeobox 1 (Drosophila) | HLX1 | 0.01 | 3.86 | 0.03 | 4.05 |  |  |  |  |  |  |  | Yes | Yes |
| **1,530** | AI201688 | Potassium voltage-gated channel, Shaw-related subfamily, member 1 | KCNC1 | 0.05 | -3.42 |  |  |  |  |  |  |  |  |  | Yes | Yes |
| **1,562** | AI992097 | Cytochrome P450, family 11, subfamily A, polypeptide 1 | CYP11A1 | 0.03 | 4.52 |  |  | 0.04 | 5.34 | -3.53 | -1.21 | -1.43 | -1.07 |  |  | Yes |
| **1,600** | AA961735 | MARCKS-like 1 | MARCKSL1 | 0.02 | 2.03 |  |  |  |  |  |  |  |  | YES |  | Yes |
| **1,601** | AI433336 | Villin 1 | VIL1 | 0.01 | -8.88 |  |  |  |  |  |  |  |  |  | Yes | Yes |
| **1,651** | AI479347 | Melanoma antigen family B, 2 | MAGEB2 | 0.03 | -4.88 |  |  |  |  |  |  |  |  |  |  | No |
| **1,753** | AI364951 | Cytochrome P450, family 4, subfamily F, polypeptide 2 | CYP4F2 | 0.03 | 4.67 |  |  |  |  |  |  |  |  |  |  | No |
| **1,808** | AA625655 | Regenerating islet-derived 1 alpha (pancreatic stone protein, pancreatic thread protein) | REG1A | 0.04 | 5.87 |  |  | 0.04 | 6.82 | -5.55 | -3.05 | -3.21 | -4.24 |  | Yes | Yes |
| **1,850** | H89996 | Transcribed locus | N/ A | 0.04 | -5.24 |  |  | 0.01 | -7.55 | -2.04 | -4.96 | -4.39 | -3.12 |  | Yes | Yes |
| **1,863** | W46900 | Chemokine (C-X-C motif) ligand 1 (melanoma growth stimulating activity, alpha) | CXCL1* | 0.02 | 4.37 | 0 | 3.36 | 0 | 13.66 | 1.73 | 5.5 | 3.85 | 7.24 |  |  | Yes |
| **1,864** | N54165 | Defensin, alpha 1 | DEFA1 | 0.03 | -5.82 |  |  | 0 | -17.89 | -3.33 | -7.46 | -5.81 | -7.77 | YES |  | Yes |
| **1,901** | AA427491 | T cell receptor alpha locus | TRA@ | 0 | 3.82 | 0 | 2.92 |  |  |  |  |  |  |  | Yes | Yes |
| **2,129** | AA706010 | Cytokine receptor-like factor 1 | CRLF1 | 0.03 | 4.46 |  |  |  |  |  |  |  |  |  | Yes | Yes |
| **2,207** | AA449336 | Protein regulator of cytokinesis | PRC1 | 0.01 | 6.9 |  |  | 0.03 | 7.22 | -5.07 | -2.5 | -2.61 | -2.34 |  | Yes | Yes |
| **2,255** | AA972293 | prostaglandin E receptor 1 (subtype EP1), 42kDa | PTGER1 | 0.01 | -8.37 |  |  |  |  |  |  |  |  |  |  | No |
| **2,282** | N49405 | Neural precursor cell expressed, developmentally down-regulated 8 | NEDD8 | 0.01 | 9.9 | 0 | 3.26 | 0 | 25.31 | -1.46 | 2.74 | 1.43 | 4.47 |  | Yes | Yes |
| **2,283** | R45111 | DAZ associated protein 2 | DAZAP2 | 0 | 2.02 |  |  |  |  |  |  |  |  |  | Yes | Yes |
| **2,285** | N62586 | Excision repair cross-complementing rodent repair deficiency, complementation group 5 | ERCC5 | 0.05 | 4.81 | 0.02 | -1.4 |  |  |  |  |  |  |  | Yes | Yes |
| **2,381** | T61271 | Phospholipase A2, group IIA (platelets, synovial fluid) | PLA2G2A | 0.01 | -6.42 |  |  |  |  |  |  |  |  |  | Yes | Yes |
| **2,432** | H08732 | Glycogen synthase 1 (muscle) | GYS1 | 0.04 | 2.67 |  |  |  |  |  |  |  |  |  | Yes | Yes |
| **2,473** | AL049611 | EF-hand domain (C-terminal) containing 1 | EFHC1 | 0.04 | -2.62 |  |  |  |  |  |  |  |  |  |  | No |
| **2,475** | N32604 | Chorionic gonadotropin, beta polypeptide | CGB | 0.03 | -5.79 | 0.02 | -6.78 |  |  |  |  |  |  |  |  | No |
| **2,489** | N93924 | Replication factor C (activator 1) 4, 37kDa | RFC4 | 0.02 | -6.52 |  |  |  |  |  |  |  |  | YES | Yes | Yes |
| **2,493** | AL163051 | Transcribed locus | N/ A | 0 | -26 |  |  |  |  |  |  |  |  |  |  | No |
| **2,501** | AA456147 | Glypican 6 | GPC6* | 0.04 | -6.46 |  |  |  |  |  |  |  |  |  |  | No |
| **2,503** | AA487912 | Guanine nucleotide binding protein (G protein), beta polypeptide 1 | GNB1 | 0.04 | 2.22 |  |  |  |  |  |  |  |  | YES | Yes | Yes |
| **2,508** | R83837 | V-yes-1 Yamaguchi sarcoma viral related oncogene homolog | LYN | 0 | 2.09 |  |  | 0.01 | 2.56 | -0.17 | 1.14 | 0.86 | 1.31 | YES | Yes | Yes |
| **2,537** | AA040170 | Chemokine (C-C motif) ligand 7 | CCL7* | 0 | 11.16 | 0 | 3.64 |  |  |  |  |  |  |  | Yes | Yes |
| **2,594** | AA035455 | Transcribed locus | N/ A | 0.04 | -6.23 |  |  | 0.01 | -5.44 | -2.04 | -4.96 | -4.39 | -3.12 |  |  | Yes |
| **2,655** | R42153 | Aspartylglucosaminidase | AGA | 0.02 | -6.3 |  |  |  |  |  |  |  |  |  | Yes | Yes |
| **2,678** | AA495835 | Erythrocyte membrane protein band 4.1-like 3 | EPB41L3 | 0 | 3.02 | 0.01 | 2.17 |  |  |  |  |  |  |  |  | No |
| **2,715** | AI017154 | Neurotensin receptor 2 | NTSR2 | 0.02 | -6.25 |  |  | 0.01 | -9.58 | -1.8 | -4.55 | -4.35 | -3.67 |  | Yes | Yes |
| **2,817** | AI889554 | Chemokine (C-X-C motif) ligand 6 (granulocyte chemotactic protein 2) | CXCL6 | 0.01 | 6.51 | 0 | 2.9 | 0.01 | 4.77 | -0.6 | 1.46 | 1.9 | 2.97 | YES | Yes | Yes |
| **2,818** | W72333 | Hypothetical protein FLJ21657 | FLJ21657 | 0.04 | -6.11 |  |  |  |  |  |  |  |  |  |  | No |
| **2,861** | AI659114 | Tubulin tyrosine ligase-like family, member 1 | TTLL1 | 0.04 | -3.88 |  |  | 0.03 | -7.81 | -2.11 | -4.76 | -4.01 | -2.99 |  | Yes | Yes |
| **2,882** | W46900 | Chemokine (C-X-C motif) ligand 1 (melanoma growth stimulating activity, alpha) | CXCL1* | 0 | 8.88 | 0.05 | 1.48 | 0 | 28.18 | 1.73 | 5.5 | 3.85 | 7.24 |  | Yes | Yes |
| **2,887** | AA398218 | Non-metastatic cells 3 | NME3 | 0.01 | 3.16 | 0 | 3.87 | 0 | 7.84 | 1.25 | 4.2 | 2.9 | 6.18 | YES | Yes | Yes |
| **3,004** | AA683578 | Adenosine deaminase | ADA | 0 | 4.2 | 0 | 3.42 | 0.05 | 3.54 | 0.29 | 1.96 | 2.19 | 3.72 | YES | Yes | Yes |
| **3,006** | AA455941 | N-acylaminoacyl-peptide hydrolase | APEH | 0.02 | 3.77 |  |  |  |  |  |  |  |  | YES | Yes | Yes |
| **3,048** | W84789 | Pregnancy-associated plasma protein A, pappalysin 1 | PAPPA | 0.04 | -2.81 |  |  |  |  |  |  |  |  |  |  | No |
| **3,068** | AA677388 | Inter-alpha (globulin) inhibitor H1 | ITIH1 | 0.02 | 6.18 |  |  |  |  |  |  |  |  |  | Yes | Yes |
| **3,084** | N63940 | Acetylcholinesterase | ACHE | 0.04 | -2.76 |  |  |  |  |  |  |  |  | YES | Yes | Yes |
| **3,101** | R00833 | Solute carrier family 2 (facilitated glucose transporter), member 2 | SLC2A2 | 0.04 | -2.09 |  |  |  |  |  |  |  |  |  | Yes | Yes |
| **3,161** | AA456314 | Tumor necrosis factor, alpha-induced protein 1 (endothelial) | TNFAIP1 | 0.01 | 6.31 | 0.02 | 3.2 |  |  |  |  |  |  |  | Yes | Yes |
| **3,230** | AI823975 | Prostaglandin E receptor 2 (subtype EP2), 53kDa | PTGER2 | 0.01 | 2.11 |  |  |  |  |  |  |  |  |  | Yes | Yes |
| **3,244** | AA703019 | RAB8B, member RAS oncogene family | RAB8B | 0.01 | 2.09 |  |  |  |  |  |  |  |  |  |  | No |
| **3,246** | N35112 | ATPase, Class V, type 10A | ATP10A | 0.01 | 3.23 |  |  |  |  |  |  |  |  | YES | Yes | Yes |
| **3,362** | NM_009588 | Lymphotoxin beta (TNF superfamily, member 3) | LTB* | 0.01 | 2.64 | 0.01 | 2.34 |  |  |  |  |  |  |  |  | No |
| **3,416** | AI767030 | Chromosome 6 open reading frame 130 | C6orf130 | 0.04 | -2.8 |  |  |  |  |  |  |  |  |  |  | No |
| **3,420** | H62864 | Chemokine (C-C motif) ligand 4-like 1 | CCL4L1* | 0.01 | 2.87 | 0 | 2.31 | 0 | 33.77 | 1.96 | 6.88 | 3.17 | 8.12 |  | Yes | Yes |
| **3,427** | R15740 | Carbohydrate (keratan sulfate Gal-6) sulfotransferase 1 | CHST1 | 0.04 | -2.41 |  |  |  |  |  |  |  |  |  | Yes | Yes |
| **3,430** | AA425102 | Chemokine (C-C motif) ligand 2 | CCL2 | 0 | 11.19 | 0 | 5.14 | 0.01 | 2.22 | 1.83 | 2.94 | 5.3 | 5.33 | YES | Yes | Yes |
| **3,440** | H00662 | Selectin L (lymphocyte adhesion molecule 1) | SELL | 0 | 2.07 | 0 | 2.39 |  |  |  |  |  |  |  | Yes | Yes |
| **3,576** | W92812 | Pro-platelet basic protein (chemokine (C-X-C motif) ligand 7) | PPBP* | 0 | 3.18 | 0.01 | 2.75 |  |  |  |  |  |  |  | Yes | Yes |
| **3,612** | AA410375 | Guanosine monophosphate reductase | GMPR | 0.01 | 3.06 | 0 | 6.21 | 0 | 3.14 | 0.35 | 1.95 | 1.92 | 4.59 | YES | Yes | Yes |
| **3,616** | R43360 | Signal recognition particle 9kDa | SRP9 | 0.04 | 4.27 |  |  |  |  |  |  |  |  |  | Yes | Yes |
| **3,652** | AA035347 | Glucosamine (N-acetyl)-6-sulfatase | GNS | 0.04 | -3.31 |  |  |  |  |  |  |  |  | YES | Yes | Yes |
| **3,668** | T81103 | Sp2 transcription factor | SP2 | 0.04 | 2.02 |  |  |  |  |  |  |  |  |  | Yes | Yes |
| **3,677** | R93176 | Carbonic anhydrase I | CA1 | 0.04 | -3.82 |  |  |  |  |  |  |  |  |  |  | No |
| **3,693** | R40790 | Gamma-aminobutyric acid (GABA) A receptor, gamma 2 | GABRG2 | 0.04 | -4.3 |  |  |  |  |  |  |  |  |  |  | No |
| **3,705** | AA778077 | Chromodomain helicase DNA binding protein 3 | CHD3 | 0.03 | 2.9 |  |  |  |  |  |  |  |  |  | Yes | Yes |
| **3,872** | AA405717 | Muscleblind-like (Drosophila) | MBNL1 | 0.04 | -5.01 |  |  |  |  |  |  |  |  |  |  | No |
| **3,927** | R43873 | Hypothetical MGC35048 | MGC35048 | 0.04 | -2.99 |  |  |  |  |  |  |  |  |  | Yes | Yes |
| **3,937** | AA486747 | V-set and immunoglobulin domain containing 4 | VSIG4 | 0.03 | -4.78 |  |  |  |  |  |  |  |  |  |  | No |
| **3,941** | AA406601 | Actin binding LIM protein 1 | ABLIM1 | 0.01 | 2.05 | 0 | 2.76 |  |  |  |  |  |  |  | Yes | Yes |
| **4,036** | AI245828 | Phosphate cytidylyltransferase 1, choline, alpha | PCYT1A | 0.04 | -4.54 |  |  |  |  |  |  |  |  |  |  | No |
| **4,077** | AW057804 | Syntaxin binding protein 2 | STXBP2 | 0.02 | -5.3 |  |  |  |  |  |  |  |  |  | Yes | Yes |
| **4,078** | AA126755 | Transcribed locus, strongly similar to XP_219265.2 PREDICTED: similar to importin 7 [Rattus norvegicus] | N/ A | 0.03 | 4.26 | 0 | 5.5 |  |  |  |  |  |  |  |  | No |
| **4,082** | AA425900 | Uracil-DNA glycosylase 2 | UNG2 | 0 | 3.66 | 0 | 3.29 | 0 | 10.56 | 1.4 | 4.79 | 3.25 | 6.53 |  | Yes | Yes |
| **4,212** | W88566 | V-raf murine sarcoma viral oncogene homolog B1 | BRAF | 0.02 | 2.11 | 0 | 5.36 |  |  |  |  |  |  |  |  | No |
| **4,217** | T49159 | Serpin peptidase inhibitor, clade B (ovalbumin), member 2 | SERPINB2 | 0.03 | 4.02 | 0 | 7.46 | 0 | 3.09 | 4.31 | 5.96 | 6.3 | 8.78 |  | Yes | Yes |
| **4,221** | H62864 | Chemokine (C-C motif) ligand 4-like 1 | CCL4L1* | 0.02 | 2.33 | 0 | 2.48 | 0 | 29.73 | 1.96 | 6.88 | 3.17 | 8.12 |  |  | Yes |
| **4,254** | AA489629 | Transcribed locus, strongly similar to NP_005737.1 pre-B-cell colony enhancing factor 1 isoform a; pre-B-cell colony-enhancing factor; 1110035O14Rik | N/ A | 0.02 | 2.33 | 0 | 2.11 | 0 | 3.2 | -2.04 | -4.96 | -4.39 | -3.12 |  | Yes | Yes |
| **4,297** | AA453293 | Phosphodiesterase 4B, cAMP-specific (phosphodiesterase E4 dunce homolog, Drosophila) | PDE4B | 0.01 | 2.88 | 0 | 2.75 | 0 | 4.27 | -2.08 | -0.17 | -0.73 | 1.24 |  | Yes | Yes |
| **4,311** | AA427595 | Src homology 2 domain containing adaptor protein B | SHB | 0.03 | 2.24 |  |  |  |  |  |  |  |  | YES | Yes | Yes |
| **4,361** | H53340 | Metallothionein 1G | MT1G | 0 | 2.55 |  |  |  |  |  |  |  |  |  |  | No |
| **4,425** | T73440 | Alanyl (membrane) aminopeptidase (aminopeptidase N, aminopeptidase M, microsomal aminopeptidase, CD13, p150) | ANPEP | 0.03 | 2.48 |  |  |  |  |  |  |  |  |  | Yes | Yes |
| **4,543** | AA779225 | Calcium channel, voltage-dependent, alpha 1H subunit | CACNA1H | 0.05 | 3.09 |  |  |  |  |  |  |  |  |  |  | No |
| **4,585** | AI084074 | B lymphoid tyrosine kinase | BLK | 0.04 | -3.6 |  |  | 0.04 | -3.64 | -1.85 | -3.05 | -3.31 | -2.46 | YES | Yes | Yes |
| **4,589** | AA018907 | Protein phosphatase 3 (formerly 2B), regulatory subunit B, 19kDa, alpha isoform (calcineurin B, type I) | PPP3R1/ CNB1 | 0.05 | 3.15 |  |  |  |  |  |  |  |  |  | Yes | Yes |
| **4,646** | AI870374 | Leucine zipper, down-regulated in cancer 1 | LDOC1 | 0.02 | -2.04 |  |  |  |  |  |  |  |  | YES | Yes | Yes |
| **4,762** | AA454668 | Prostaglandin-endoperoxide synthase 1 (prostaglandin G/H synthase and cyclooxygenase) | PTGS1* | 0 | 2.06 |  |  |  |  |  |  |  |  |  | Yes | Yes |
| **4,806** | AA666269 | Integrin, beta 3 (platelet glycoprotein IIIa, antigen CD61) | ITGB3 | 0.04 | 3.48 |  |  |  |  |  |  |  |  |  | Yes | Yes |
| **4,808** | N80129 | Metallothionein 1X | MT1X | 0.03 | 3.56 | 0.04 | 2.24 |  |  |  |  |  |  |  |  | No |
| **4,810** | AA485052 | Proteasome (prosome, macropain) 26S subunit, non-ATPase, 3 | PSMD3 | 0.01 | 3.87 |  |  |  |  |  |  |  |  |  | Yes | Yes |
| **4,862** | N95761 | Fucosidase, alpha-L- 1, tissue | FUCA1 | 0 | 4.53 |  |  | 0 | -2.25 | 3.32 | 2.16 | 5.53 | 2.93 |  | Yes | Yes |
| **4,863** | N/A | Spondin 1, extracellular matrix protein | SPON1 | 0.04 | -4.31 |  |  |  |  |  |  |  |  |  | Yes | Yes |
| **4,910** | AA521026 | 8-oxoguanine DNA glycosylase | OGG1 | 0 | -7.28 |  |  | 0.04 | -3.56 | -3.49 | -5.1 | -5.85 | -4.16 |  | Yes | Yes |
| **4,919** | H86558 | MAX dimerization protein 1 | MXD1 | 0 | 2.87 | 0 | 1.94 |  |  |  |  |  |  | YES |  | Yes |
| **4,971** | AA455955 | Proprotein convertase subtilisin/kexin type 7 | PCSK7 | 0.01 | 2.13 |  |  |  |  |  |  |  |  | YES | Yes | Yes |
| **5,120** | AI311734 | Fatty acid binding protein 6, ileal (gastrotropin) | FABP6 | 0.02 | -3.04 |  |  |  |  |  |  |  |  | YES | Yes | Yes |
| **5,156** | AA258396 | Pleckstrin homology-like domain, family A, member 1 | PHLDA1* | 0.02 | 3.02 |  |  |  |  |  |  |  |  |  | Yes | Yes |
| **5,197** | AW003591 | Triosephosphate isomerase 1 | TPI1 | 0.04 | -2.76 | 0.03 | 2.36 |  |  |  |  |  |  |  |  | No |
| **5,339** | R77718 | Casein kinase 1, alpha 1 | CSNK1A1 | 0 | 2 |  |  |  |  |  |  |  |  |  |  | No |
| **5,357** | H11003 | Endothelin 1 | EDN1 | 0.02 | -2.13 | 0.01 | 1.85 |  |  |  |  |  |  |  |  | No |
| **5,438** | R53935 | ATP-binding cassette, sub-family B (MDR/TAP), member 4 | ABCB4 | 0.03 | -3.39 |  |  |  |  |  |  |  |  |  |  | No |
| **5,462** | R60313 | CDNA clone IMAGE:5276765 | N/ A | 0 | 2.19 |  |  |  |  |  |  |  |  |  |  | No |
| **5,604** | AA428196 | POU domain, class 4, transcription factor 1 | POU4F1 | 0.04 | -3.22 |  |  | 0.02 | -4.41 | -1.85 | -3.49 | -3.13 | -2.36 |  | Yes | Yes |
| **5,611** | R40400 | Cell adhesion molecule with homology to L1CAM (close homolog of L1) | CHL1 | 0.01 | -4.7 |  |  |  |  |  |  |  |  |  |  | No |
| **5,713** | AA480876 | Activator of basal transcription 1 | ABT1 | 0 | 4.01 |  |  |  |  |  |  |  |  |  | Yes | Yes |
| **5,718** | AA056093 | Rhodopsin (opsin 2, rod pigment) (retinitis pigmentosa 4, autosomal dominant) | RHO | 0.04 | -2.5 |  |  |  |  |  |  |  |  |  |  | No |
| **5,762** | AI815076 | Solute carrier family 7 (cationic amino acid transporter, y+ system), member 7 | SLC7A7 | 0.01 | 2.3 | 0.02 | 2.06 | 0.01 | 2.03 | 0.71 | 1.7 | 1.87 | 2.72 |  | Yes | Yes |
| **5,774** | AA446246 | Neuroblastoma RAS viral (v-ras) oncogene homolog | NRAS | 0.01 | 3.37 |  |  |  |  |  |  |  |  | YES | Yes | Yes |
| **5,781** | AI523637 | Protocadherin 9 | PCDH9 | 0.04 | 2.33 | 0.01 | 2.45 |  |  |  |  |  |  |  | Yes | Yes |
| **5,801** | AI473336 | WNT1 inducible signaling pathway protein 1 | WISP1 | 0.05 | -2.26 |  |  |  |  |  |  |  |  |  |  | No |
| **5,822** | AC009246 | Protein geranylgeranyltransferase type I, beta subunit | PGGT1B | 0.01 | -5.71 |  |  |  |  |  |  |  |  |  |  | No |
| **6,003** | AA279429 | Endothelin converting enzyme 1 | ECE1 | 0.03 | 2.03 |  |  |  |  |  |  |  |  |  | Yes | Yes |
| **6,046** | H44032 | Gap junction protein, alpha 4, 37kDa (connexin 37) | GJA4 | 0.03 | -2.82 |  |  |  |  |  |  |  |  |  |  | No |
| **6,208** | AA167728 | CD27-binding (Siva) protein | SIVA | 0.03 | -2.87 |  |  |  |  |  |  |  |  |  |  | No |
| **6,305** | AA086038 | Phospholipase A2 receptor 1, 180kDa | PLA2R1 | 0.01 | -2.94 |  |  |  |  |  |  |  |  |  | Yes | Yes |
| **6,328** | AA461485 | Leukocyte cell derived chemotaxin 1 | LECT1 | 0 | 3.12 | 0 | 1.94 |  |  |  |  |  |  |  |  | No |
| **6,329** | AA609992 | Dehydrogenase/reductase (SDR family) member 9 | DHRS9 | 0.02 | -3.57 | 0 | -4.14 | 0 | -2.03 | -0.48 | -1.47 | -2 | -3.13 |  |  | Yes |
| **6,533** | AI659563 | Androgen receptor (dihydrotestosterone receptor; testicular feminization; spinal and bulbar muscular atrophy; Kennedy disease) | AR | 0.05 | -3 |  |  | 0.02 | -2.7 | -2.78 | -3.9 | -4.16 | -2.77 |  | Yes | Yes |
| **6,534** | H38799 | Hypothetical protein FLJ20701 | FLJ20701 | 0.01 | 2.48 |  |  |  |  |  |  |  |  |  |  | No |
| **6,620** | AA464711 | Complement component 3a receptor 1 | C3AR1 | 0.01 | 2.07 |  |  |  |  |  |  |  |  | YES | Yes | Yes |
| **6,687** | N29376 | Myeloid cell nuclear differentiation antigen | MNDA | 0.01 | -2.49 | 0 | -2.11 | 0 | -3 | 3.55 | 1.97 | 2.23 | 0.92 | YES | Yes | Yes |
| **6,714** | AA872383 | Metallothionein 2A | MT2A | 0 | 2.1 | 0 | 2.35 |  |  |  |  |  |  |  |  | No |
| **6,836** | AA401693 | CD163 molecule | CD163* | 0 | 3.13 |  |  |  |  |  |  |  |  | YES | Yes | Yes |
| **6,899** | AA884281 | Syntaxin 11 | STX11 | 0.01 | -3.28 |  |  |  |  |  |  |  |  |  | Yes | Yes |
| **7,012** | AI924973 | Killer cell lectin-like receptor subfamily B, member 1 | KLRB1 | 0 | -2.14 |  |  |  |  |  |  |  |  |  | Yes | Yes |
| **7,099** | H09818 | Cytoplasmic FMR1 interacting protein 2 | CYFIP2 | 0 | 2.07 | 0 | 2.08 |  |  |  |  |  |  | YES |  | Yes |
| **7,182** | AA890663 | P21/Cdc42/Rac1-activated kinase 1 (STE20 homolog, yeast) | PAK1 | 0.04 | 2.02 |  |  |  |  |  |  |  |  |  | Yes | Yes |
| **7,226** | R60343 | 5'-nucleotidase, ecto (CD73) | NT5E | 0.01 | 3.79 |  |  |  |  |  |  |  |  |  | Yes | Yes |
| **7,331** | AA456298 | Histone 2, H2be | HIST2H2BE | 0 | 2.72 |  |  |  |  |  |  |  |  |  |  | No |
| **7,428** | H77597 | Metallothionein 1H | MT1H | 0.01 | 2.18 | 0.02 | 2.61 |  |  |  |  |  |  | YES | Yes | Yes |
| **7,510** | AI351740 | Lymphotoxin beta (TNF superfamily, member 3) | LTB* | 0.02 | 2.11 | 0.02 | 2.3 |  |  |  |  |  |  |  |  | No |
| **7,520** | AI341924 | Transcribed locus | N/ A | 0.05 | -2.9 |  |  |  |  |  |  |  |  |  |  | No |
| **7,617** | AC019206 | Farnesyltransferase, CAAX box, beta | FNTB | 0 | 2.21 | 0.03 | 1.8 |  |  |  |  |  |  |  | Yes | Yes |
| **7,722** | N98591 | Interleukin 6 (interferon, beta 2) | IL6 | 0.01 | 3.25 | 0 | 13.12 | 0 | 18.74 | 0.21 | 4.38 | 1.88 | 8.06 |  | Yes | Yes |
| **7,745** | AA599158 | Glutamyl-prolyl-tRNA synthetase | EPRS | 0 | 2.9 | 0.01 | 2.19 |  |  |  |  |  |  |  | Yes | Yes |
| **7,755** | AA040170 | Chemokine (C-C motif) ligand 7 | CCL7* | 0 | 7.89 | 0.01 | 3.11 |  |  |  |  |  |  |  | Yes | Yes |
| **7,781** | R33642 | Glutathione S-transferase pi | GSTP1 | 0.03 | -3.17 |  |  |  |  |  |  |  |  | YES | Yes | Yes |
| **7,809** | AA843718 | General transcription factor IIIC, polypeptide 1, alpha 220kDa | GTF3C1/ TFIIIC | 0.02 | 3.98 |  |  | 0.04 | 3.8 | -1.93 | -0.36 | -0.3 | -0.23 | YES |  | Yes |
| **7,838** | N67487 | Microfibrillar-associated protein 2 | MFAP2 | 0 | -2.1 |  |  |  |  |  |  |  |  |  |  | No |
| **7,853** | AA018591 | Spectrin, beta, non-erythrocytic 1 | SPTBN1 | 0.01 | 4.78 | 0 | 2.85 | 0 | 17.21 | 2.45 | 6.56 | 4.69 | 8.02 |  | Yes | Yes |
| **8,021** | AC068288 | Jumonji domain containing 1A | JMJD1A | 0.01 | 3.45 |  |  |  |  |  |  |  |  |  |  | No |
| **8,035** | AI640779 | Solute carrier family 12 (sodium/chloride transporters), member 3 | SLC12A3 | 0.03 | -3.06 |  |  | 0.01 | -3.33 | -1.62 | -2.92 | -2.93 | -2.89 |  | Yes | Yes |
| **8,130** | AI285199 | Chemokine (C-C motif) ligand 20 | CCL20 | 0 | 10.19 | 0 | 4.52 | 0 | 74.79 | 0.23 | 6.41 | 3.54 | 8.49 |  | Yes | Yes |
| **8,175** | AI189606 | Leukocyte-associated immunoglobulin-like receptor 2 | LAIR2 | 0.04 | 2.02 |  |  |  |  |  |  |  |  | YES | Yes | Yes |
| **8,281** | AA449440 | Interferon gamma receptor 2 (interferon gamma transducer 1) | IFNGR2 | 0 | 2.01 |  |  | 0 | 2.97 | 1.81 | 3.36 | 2.79 | 3.33 | YES | Yes | Yes |
| **8,357** | AA935273 | Chemokine (C-X-C motif) ligand 3 | CXCL3* | 0.02 | 3.72 | 0 | 3 | 0 | 9.8 | 2.87 | 6.19 | 4.78 | 7.74 |  | Yes | Yes |
| **8,732** | T60082 | CXXC finger 1 (PHD domain) | CXXC1 | 0 | 2.24 |  |  |  |  |  |  |  |  |  |  | No |
| **8,747** | N57526 | Amyloid beta (A4) precursor protein-binding, family B, member 3 | APBB3 | 0 | 2.1 |  |  |  |  |  |  |  |  |  |  | No |
| **8,889** | R19189 | C-type lectin domain family 12, member A | CLEC12A | 0 | -2.15 |  |  |  |  |  |  |  |  |  |  | No |
| **8,983** | AF131889 | chlamydia pneumoniae momp gene | N/ A | 0.02 | -3.14 |  |  |  |  |  |  |  |  |  |  | No |
| **9,012** | AA598652 | Ribosomal protein L39-like | RPL39L | 0 | 2.54 | 0 | 2.3 |  |  |  |  |  |  |  |  | No |
| **9,036** | R52542 | IMP (inosine monophosphate) dehydrogenase 1 | IMPDH1 | 0.01 | -3.24 |  |  | 0.01 | -2.22 | -1.92 | -2.85 | -3.16 | -2.45 |  | Yes | Yes |
| **9,060** | R45255 | EBNA1 binding protein 2 | EBNA1BP2 | 0.05 | -2.48 |  |  |  |  |  |  |  |  |  |  | No |
| **9,068** | AA633835 | Lipoprotein lipase | LPL | 0.03 | -2.2 | 0.03 | -2.35 |  |  |  |  |  |  |  | Yes | Yes |
| **9,564** | AA046424 | Acyl-CoA thioesterase 4 | ACOT4 | 0.04 | -2.73 |  |  |  |  |  |  |  |  |  |  | No |
| **9,588** | AA059378 | Guanylate cyclase activator 1A (retina) | GUCA1A | 0.05 | 2.08 |  |  |  |  |  |  |  |  |  |  | No |
| **9,623** | T98266 | ESTs | N/ A | 0.04 | 7.48 |  |  |  |  |  |  |  |  |  |  | No |
| **9,653** | N70196 | Cingulin | CGN | 0 | -13.53 |  |  |  |  |  |  |  |  |  |  | No |
| **9,731** | AA131885 | Hypothetical protein LOC51057 | LOC51057 | 0.01 | -7.59 |  |  | 0.04 | -5.18 | -1.42 | -3.81 | -4.25 | -1.75 |  |  | Yes |
| **9,834** | AA058709 | Formyl peptide receptor-like 2 | FPRL2 | 0.01 | 2.07 |  |  |  |  |  |  |  |  | YES |  | Yes |
| **9,937** | R37145 | RAD51 homolog C | RAD51C | 0.04 | 4.3 |  |  |  |  |  |  |  |  |  |  | No |
| **9,973** | R51506 | Potassium channel, subfamily T, member 2 | KCNT2 | 0.04 | 6.19 |  |  |  |  |  |  |  |  |  |  | No |
| **9,984** | R72517 | Transcribed locus | N/ A | 0 | 2.04 |  |  |  |  |  |  |  |  |  |  | No |
| **10,069** | AA430409 | Multidrug resistance-related protein | MGC13170 | 0.04 | 5.33 |  |  |  |  |  |  |  |  |  |  | No |
| **10,086** | AA707066 | Transcribed locus | N/ A | 0.02 | -5.47 |  |  |  |  |  |  |  |  |  |  | No |
| **10,107** | T65118 | Catenin (cadherin-associated protein), alpha 1, 102kDa | CTNNA1 | 0.03 | 2.75 |  |  |  |  |  |  |  |  |  |  | No |
| **10,140** | W46900 | Chemokine (C-X-C motif) ligand 1 (melanoma growth stimulating activity, alpha) | CXCL1* | 0.01 | 5.23 | 0 | 2.81 | 0 | 15.44 | 1.73 | 5.5 | 3.85 | 7.24 |  |  | Yes |
| **10,150** | AA404239 | Enabled homolog (Drosophila) | ENAH | 0.01 | 3.28 | 0 | 3.38 | 0 | 8.56 | 0.94 | 4.06 | 2.66 | 5.84 |  | Yes | Yes |
| **10,193** | R90744 | Chromosome 1 open reading frame 86 | C1orf86 | 0.01 | -6.7 |  |  |  |  |  |  |  |  |  |  | No |
| **10,405** | N21237 | SAM and SH3 domain containing 1 | SASH1 | 0 | 2.24 | 0 | 2.49 |  |  |  |  |  |  |  |  | No |
| **10,515** | H65596 | Sin3A-associated protein, 18kDa | SAP18 | 0.05 | 3.23 |  |  |  |  |  |  |  |  |  | Yes | Yes |
| **10,553** | H96508 | Transcribed locus | N/ A | 0.03 | 6.53 |  |  |  |  |  |  |  |  |  |  | No |
| **10,646** | N23882 | Transcribed locus | N/ A | 0.03 | -3.77 |  |  |  |  |  |  |  |  |  |  | No |
| **10,688** | R32836 | Transcribed locus | N/ A | 0.01 | -9.93 |  |  |  |  |  |  |  |  |  |  | No |
| **10,710** | H61243 | Uncoupling protein 2 (mitochondrial, proton carrier) | UCP2 | 0 | 2.67 |  |  |  |  |  |  |  |  |  | Yes | Yes |
| **10,720** | N55459 | Metallothionein 1F (functional) | MT1F | 0.02 | 3.43 | 0.01 | 3.38 |  |  |  |  |  |  | YES |  | Yes |
| **10,727** | AA101971 | V-ets erythroblastosis virus E26 oncogene homolog 1 (avian) | ETS1 | 0 | 4.2 | 0 | 3.21 |  |  |  |  |  |  | YES | Yes | Yes |
| **10,766** | AA489068 | Cytokine-like nuclear factor n-pac | N-PAC | 0.01 | 4.59 | 0 | 3.35 | 0 | 2.39 | 4.66 | 5.92 | 6.82 | 7.6 |  |  | Yes |
| **10,767** | R38645 | Chromosome X open reading frame 36 | CXorf36 | 0.02 | -4.59 |  |  | 0.04 | -4.59 | -6.8 | -8.97 | -8.97 | -8.97 |  |  | Yes |
| **10,937** | W02699 | Tumor necrosis factor (ligand) superfamily, member 8 | TNFSF8 | 0.04 | 6.3 |  |  |  |  |  |  |  |  |  |  | No |
| **11,038** | N71796 | Fc receptor-like and mucin-like 1 | FCRLM1 | 0.04 | -7.9 |  |  | 0.01 | -12.24 | -1.66 | -5.3 | -4.64 | -3.59 |  |  | Yes |
| **11,052** | H11453 | Transcribed locus | N/ A | 0.05 | 3.29 |  |  |  |  |  |  |  |  |  |  | No |
| **11,060** | H99460 | Transient receptor potential cation channel, A, member 1 | TRPA1 | 0.01 | 2.05 |  |  |  |  |  |  |  |  |  |  | No |
| **11,061** | H59780 | Heparan sulfate (glucosamine) 3-O-sulfotransferase 3B1 | HS3ST3B1* | 0.01 | 2.84 | 0 | 2.7 | 0 | 6.19 | 1.46 | 4.13 | 2.99 | 5.6 |  |  | Yes |
| **11,082** | H09748 | B-cell CLL/lymphoma 11B | BCL11B | 0 | 2.06 |  |  |  |  |  |  |  |  |  |  | No |
| **11,084** | N34415 | FERM domain containing 4B | FRMD4B | 0.01 | -7.85 | 0.05 | 6.34 | 0 | -15.85 | -2.83 | -6.96 | -5.96 | -4.32 |  |  | Yes |
| **11,120** | N21228 | Nudix (nucleoside diphosphate linked moiety X)-type motif 21 | NUDT21 | 0.03 | -3.8 |  |  |  |  |  |  |  |  |  |  | No |
| **11,134** | N34839 | N/A | N/ A | 0.03 | -9.38 |  |  |  |  |  |  |  |  |  |  | No |
| **11,173** | R54416 | MAWD binding protein | MAWBP | 0.02 | -11.39 |  |  |  |  |  |  |  |  |  |  | No |
| **11,190** | N51614 | Formin-like 1 | FMNL1 | 0.03 | 4.11 |  |  |  |  |  |  |  |  |  |  | No |
| **11,291** | AA703609 | Glypican 6 | GPC6* | 0.02 | -10.02 | 0 | -22.15 |  |  |  |  |  |  |  |  | No |
| **11,319** | N29914 | Endothelin receptor type B | EDNRB | 0.04 | 6.55 |  |  | 0.01 | 11.64 | -6.78 | -3.25 | -4.06 | -5.11 |  | Yes | Yes |
| **11,370** | AA156781 | Embigin homolog (mouse) | EMB | 0.02 | 2.21 |  |  |  |  |  |  |  |  |  |  | No |
| **11,391** | AA682780 | ESTs | N/ A | 0.02 | -6.03 |  |  |  |  |  |  |  |  |  |  | No |
| **-11,394** | W52182 | LRP16 protein | LRP16 | 0.01 | -8.98 |  |  |  |  |  |  |  |  |  |  | No |
| **11,507** | R07749 | Copine III | CPNE3 | 0.03 | -9.68 |  |  |  |  |  |  |  |  |  |  | No |
| **11,566** | H28681 | Delta-notch-like EGF repeat-containing transmembrane | DNER | 0.01 | 2.52 |  |  |  |  |  |  |  |  |  |  | No |
| **11,632** | H51425 | Full length insert cDNA YN73H08 | N/ A | 0.02 | -4.07 |  |  |  |  |  |  |  |  |  |  | No |
| **11,665** | H65832 | O-acyltransferase (membrane bound) domain containing 5 | OACT5 | 0.01 | 9.87 |  |  |  |  |  |  |  |  |  |  | No |
| **11,678** | H99490 | MAM domain containing glycosylphosphatidylinositol anchor 1 | MDGA1 | 0.04 | -5.31 |  |  | 0.02 | -7.45 | -6.1 | -8.97 | -8.48 | -8.97 |  |  | Yes |
| **11,740** | W42459 | RecQ protein-like (DNA helicase Q1-like) | RECQL | 0.03 | -4.26 |  |  |  |  |  |  |  |  |  |  | No |
| **11,744** | AA406059 | EST | N/ A | 0.03 | -7.82 |  |  | 0.04 | -4.22 | -2.04 | -4.96 | -4.39 | -3.12 |  |  | Yes |
| **11,745** | R96337 | HEAT repeat containing 1 | HEATR1 | 0 | 16.6 |  |  |  |  |  |  |  |  |  |  | No |
| **11,754** | AA609904 | Chromosome 17 open reading frame 75 | C17orf75 | 0 | 10.4 |  |  |  |  |  |  |  |  |  |  | No |
| **11,788** | R82317 | SMAD, mothers against DPP homolog 5 (Drosophila) | SMAD5 | 0.03 | -7.21 |  |  |  |  |  |  |  |  |  |  | No |
| **11,814** | N23174 | Solute carrier family 7 (cationic amino acid transporter, y+ system), member 8 | SLC7A8 | 0.02 | 5.74 |  |  |  |  |  |  |  |  |  | Yes | Yes |
| **11,864** | N24538 | NOL1/NOP2/Sun domain family, member 2 | NSUN2 | 0.05 | 5.69 |  |  |  |  |  |  |  |  |  |  | No |
| **11,884** | W86282 | Rho family GTPase 3 | RND3 | 0.04 | -6.99 |  |  | 0.03 | -6.56 | -2.25 | -4.95 | -5.05 | -5.26 |  |  | Yes |
| **11,922** | AA974848 | Dystrophia myotonica-protein kinase | DMPK | 0.01 | 4.16 | 0.02 | 3.35 |  |  |  |  |  |  |  | Yes | Yes |
| **11,946** | AA489211 | Signal transducing adaptor molecule (SH3 domain and ITAM motif) 2 | STAM2 | 0.04 | 3.13 |  |  |  |  |  |  |  |  |  |  | No |
| **11,947** | AA704946 | Paf1, RNA polymerase II associated factor, homolog (S. cerevisiae) | PAF1 | 0.03 | 6.47 |  |  |  |  |  |  |  |  |  |  | No |
| **11,956** | AA489224 | PHD finger protein 10 | PHF10 | 0.05 | 2.61 | 0.04 | 2.86 |  |  |  |  |  |  |  |  | No |
| **11,964** | N47443 | Zinc finger protein 536 | ZNF536 | 0.01 | -8.2 |  |  | 0.03 | -6.82 | -2.27 | -5.08 | -5.34 | -3.76 |  |  | Yes |
| **11,994** | AA027230 | Exportin 6 | XPO6 | 0.02 | 4.75 |  |  |  |  |  |  |  |  |  |  | No |
| **12,158** | N35889 | Transcribed locus | N/ A | 0.01 | -2.24 | 0.03 | -1.49 |  |  |  |  |  |  |  |  | No |
| **12,174** | N73680 | Solute carrier family 11 (proton-coupled divalent metal ion transporters), member 2 | SLC11A2/ NRAMP2 | 0 | 2.01 | 0 | 1.81 | 0 | 2.53 | 1.35 | 2.7 | 2.36 | 3.57 |  | Yes | Yes |
| **12,182** | AA063573 | SAM domain, SH3 domain and nuclear localisation signals, 1 | SAMSN1* | 0.01 | 2.39 | 0 | 1.97 | 0.01 | 2.14 | 3.27 | 4.4 | 4.56 | 5.4 |  | Yes | Yes |
| **12,272** | H18663 | Follistatin-like 4 | FSTL4 | 0.02 | -4.57 |  |  |  |  |  |  |  |  |  |  | No |
| **12,374** | H26271 | Pleckstrin homology-like domain, family A, member 1 | PHLDA1* | 0.02 | 3.78 |  |  |  |  |  |  |  |  |  | Yes | Yes |
| **12,423** | R60705 | Hairy/enhancer-of-split related with YRPW motif 1 | HEY1 | 0.05 | -3.15 |  |  |  |  |  |  |  |  |  | Yes | Yes |
| **12,460** | N51296 | U2-associated SR140 protein | SR140 | 0 | -9.38 | 0.02 | -7.23 |  |  |  |  |  |  |  |  | No |
| **12,496** | AA706804 | Methyl-CpG binding domain protein 3 | MBD3 | 0.01 | -6.38 |  |  |  |  |  |  |  |  |  |  | No |
| **12,565** | R76554 | Calmodulin 1 (phosphorylase kinase, delta) | CALM1 | 0 | 2 |  |  |  |  |  |  |  |  |  |  | No |
| **12,599** | AA459402 | Mov10, Moloney leukemia virus 10, homolog (mouse) | MOV10 | 0.03 | 6.81 |  |  |  |  |  |  |  |  |  |  | No |
| **12,716** | W92812 | Pro-platelet basic protein (chemokine (C-X-C motif) ligand 7) | PPBP* | 0.02 | 2.63 | 0.01 | 2.37 |  |  |  |  |  |  |  | Yes | Yes |
| **12,814** | W88725 | Melanoma antigen family D, 1 | MAGED1 | 0 | -2.13 |  |  |  |  |  |  |  |  |  |  | No |
| **12,859** | AA010224 | Hypothetical protein LOC283663 | LOC283663 | 0 | 2.8 | 0 | 2.09 |  |  |  |  |  |  |  |  | No |
| **12,926** | R39364 | Clones 24632 and 24634 mRNA sequence | N/ A | 0.04 | -4.12 |  |  | 0.03 | -4.88 | -2.04 | -4.96 | -4.39 | -3.12 |  |  | Yes |
| **12,985** | AA700041 | Transcribed locus | N/ A | 0.02 | -8.53 |  |  |  |  |  |  |  |  |  |  | No |
| **12,986** | N30680 | STEAP family member 4 | STEAP4 | 0.04 | 3.66 |  |  |  |  |  |  |  |  |  |  | No |
| **13,003** | R32893 | Transcribed locus | N/ A | 0.03 | 6.44 |  |  |  |  |  |  |  |  |  |  | No |
| **13,030** | AA703219 | Dedicator of cytokinesis 10 | DOCK10 | 0.03 | 2.06 |  |  |  |  |  |  |  |  |  |  | No |
| **13,043** | R33402 | SAM domain, SH3 domain and nuclear localisation signals, 1 | SAMSN1* | 0 | 2.84 | 0 | 2.38 |  |  |  |  |  |  |  | Yes | Yes |
| **13,068** | H00982 | Ubiquitin specific peptidase 40 | USP40 | 0.01 | 7.59 | 0.02 | -7.18 |  |  |  |  |  |  |  |  | No |
| **13,072** | AA610043 | Synaptotagmin-like 2 | SYTL2 | 0.01 | -8.12 |  |  |  |  |  |  |  |  |  |  | No |
| **13,087** | AA705264 | F-box protein 4 | FBXO4 | 0.02 | 7.48 | 0.02 | -7.79 |  |  |  |  |  |  |  |  | No |
| **13,123** | T90871 | Transcribed locus | N/ A | 0.03 | 5.45 |  |  |  |  |  |  |  |  |  |  | No |
| **13,137** | AA682381 | SEC13-like 1 (S. cerevisiae) | SEC13L1 | 0.04 | -4.88 |  |  |  |  |  |  |  |  |  | Yes | Yes |
| **13,157** | AA682407 | Transcribed locus | N/ A | 0.02 | -5.92 |  |  | 0.05 | -3.82 | -2.04 | -4.96 | -4.39 | -3.12 |  |  | Yes |
| **13,192** | AA973337 | PCF11, cleavage and polyadenylation factor subunit, homolog (S. cerevisiae) | PCF11 | 0 | -9.75 |  |  | 0.04 | -3.87 | -2.03 | -3.97 | -5.16 | -4.73 |  | Yes | Yes |
| **13,339** | W47171 | Zinc finger protein 302 | ZNF302 | 0.04 | -4.03 |  |  |  |  |  |  |  |  |  |  | No |
| **13,454** | N36083 | Glycosyltransferase-like domain containing 1 | GTDC1 | 0.03 | -6.32 |  |  | 0.02 | -11.08 | -3.88 | -7.41 | -6.61 | -5.09 |  |  | Yes |
| **13,479** | W95636 | Glypican 4 | GPC4 | 0.05 | -5.75 |  |  |  |  |  |  |  |  | YES | Yes | Yes |
| **13,496** | AA676604 | Mortality factor 4 like 2 | MORF4L2 | 0 | 14.28 |  |  | 0 | 13.39 | -6.32 | -2.67 | -2.6 | -3.29 |  |  | Yes |
| **13,510** | AA132065 | Transcribed locus | N/ A | 0.01 | 4.63 |  |  | 0.04 | 3.93 | -2.04 | -4.96 | -4.39 | -3.12 |  |  | Yes |
| **13,539** | W74696 | Transcribed locus | N/ A | 0.02 | 5.79 | 0.01 | 10.77 |  |  |  |  |  |  |  |  | No |
| **13,548** | AA487054 | Transcribed locus | N/ A | 0.04 | -2.1 |  |  |  |  |  |  |  |  |  |  | No |
| **13,621** | H90744 | Protein tyrosine phosphatase, receptor type, G | PTPRG | 0.02 | 7.52 |  |  |  |  |  |  |  |  |  | Yes | Yes |
| **13,629** | H99058 | Chromosome 1 open reading frame 149 | C1orf149 | 0 | 2.19 |  |  |  |  |  |  |  |  |  |  | No |
| **13,675** | N48348 | CDNA clone IMAGE:4794876 | N/ A | 0 | -14.65 |  |  |  |  |  |  |  |  |  |  | No |
| **13,693** | T56021 | Carboxypeptidase D | CPD | 0.02 | -8.44 |  |  |  |  |  |  |  |  |  | Yes | Yes |
| **13,746** | AA707195 | FLJ36874 protein | FLJ36874 | 0.02 | -4.51 |  |  |  |  |  |  |  |  |  |  | No |
| **13,757** | R49305 | Glycogenin 2 | GYG2 | 0.01 | -8.8 |  |  |  |  |  |  |  |  |  | Yes | Yes |
| **13,772** | AA279628 | Membrane-spanning 4-domains, subfamily A, member 1 | MS4A1 | 0 | 3.7 | 0 | 2.67 |  |  |  |  |  |  |  |  | No |
| **14,009** | AA464979 | Hypothetical protein FLJ21820 | FLJ21820 | 0.03 | 4.5 |  |  |  |  |  |  |  |  |  |  | No |
| **14,031** | W95041 | Heparan sulfate (glucosamine) 3-O-sulfotransferase 3B1 | HS3ST3B1* | 0.01 | 3 | 0 | 3.52 | 0 | 5.47 | 1.46 | 4.13 | 2.99 | 5.6 |  | Yes | Yes |
| **14,225** | AA701448 | Protein tyrosine phosphatase-like (proline instead of catalytic arginine), member A | PTPLA | 0.05 | 6.07 |  |  |  |  |  |  |  |  |  |  | No |
| **14,248** | H02039 | Melanoma associated antigen (mutated) 1-like 1 | MUM1L1 | 0.04 | -6.14 |  |  |  |  |  |  |  |  |  |  | No |
| **14,265** | AA705684 | SH3-domain binding protein 1 | SH3BP1 | 0.04 | -6.54 |  |  |  |  |  |  |  |  |  |  | No |
| **14,302** | AA972350 | Surfactant, pulmonary-associated protein B | SFTPB | 0.02 | -5.4 |  |  | 0.03 | -5.65 | -2.61 | -4.82 | -5.02 | -3.55 |  | Yes | Yes |
| **14,303** | H79148 | Tetratricopeptide repeat domain 22 | TTC22 | 0.03 | -7.48 |  |  |  |  |  |  |  |  |  |  | No |
| **14,360** | AA401477 | Transcribed locus | N/ A | 0.05 | -3.8 |  |  | 0.03 | -5.09 | -2.04 | -4.96 | -4.39 | -3.12 |  |  | Yes |
| **14,388** | R67373 | Transcribed locus | N/ A | 0 | -12.7 |  |  |  |  |  |  |  |  |  |  | No |
| **14,664** | N40949 | Transcribed locus, weakly similar to XP_342242.2 PREDICTED: similar to LRRG00116 [Rattus norvegicus] | N/ A | 0.03 | -5.6 |  |  |  |  |  |  |  |  |  |  | No |
| **14,666** | AA680186 | Chemokine (C-C motif) ligand 19 | CCL19 | 0.02 | -9.16 |  |  |  |  |  |  |  |  |  | Yes | Yes |
| **14,677** | H15417 | Glutamate receptor, ionotropic, kainate 2 | GRIK2 | 0.04 | 6.34 |  |  |  |  |  |  |  |  |  | Yes | Yes |
| **14,702** | R44581 | Transcribed locus | N/ A | 0.04 | -4.61 |  |  |  |  |  |  |  |  |  |  | No |
| **14,707** | H10403 | Protein tyrosine phosphatase, receptor type, D | PTPRD | 0.05 | -2.48 |  |  |  |  |  |  |  |  |  |  | No |
| **14,802** | N59214 | ATP-binding cassette, sub-family G (WHITE), member 2 | ABCG2 | 0.01 | -9 |  |  |  |  |  |  |  |  |  |  | No |
| **14,894** | N75498 | Chromosome 16 open reading frame 45 | C16orf45 | 0 | -11.84 |  |  | 0.04 | -4.56 | -2.84 | -4.85 | -6.41 | -4.41 |  |  | Yes |
| **14,912** | AA282236 | KIAA1128 | KIAA1128 | 0.03 | 8.15 |  |  |  |  |  |  |  |  |  | Yes | Yes |
| **14,947** | AA883236 | Membrane associated guanylate kinase, WW and PDZ domain containing 2 | MAGI2 | 0.03 | -4.3 |  |  |  |  |  |  |  |  |  |  | No |
| **14,959** | W33021 | Interferon regulatory factor 2 | IRF2 | 0 | -16.3 | 0.01 | -11.83 |  |  |  |  |  |  |  | Yes | Yes |
| **15,423** | R36112 | T-complex-associated-testis-expressed 3 | TCTE3 | 0.03 | 6.67 |  |  |  |  |  |  |  |  |  |  | No |
| **15,450** | AA777510 | Ribosomal protein S6 kinase, 90kDa, polypeptide 2 | RPS6KA2 | 0.01 | -7.1 | 0 | -10.7 |  |  |  |  |  |  |  |  | No |
| **15,502** | AA953294 | Sjogren's syndrome nuclear autoantigen 1 | SSNA1 | 0.03 | 3.94 |  |  |  |  |  |  |  |  |  |  | No |
| **15,516** | AA521384 | Solute carrier family 39 (zinc transporter), member 8 | SLC39A8 | 0 | 4.92 |  |  | 0 | 11 | -2.09 | 1.35 | 0.21 | 1.66 | YES |  | Yes |
| **15,520** | H11938 | MYST histone acetyltransferase 2 | MYST2 | 0.02 | -5.09 |  |  |  |  |  |  |  |  |  |  | No |
| **15,554** | H15662 | General transcription factor II, i | GTF2I | 0.01 | 10.56 |  |  |  |  |  |  |  |  |  | Yes | Yes |
| **15,557** | AA699882 | Steroid receptor RNA activator 1 | SRA1 | 0.04 | 6.56 |  |  |  |  |  |  |  |  |  |  | No |
| **15,591** | AA459008 | Transcribed locus | N/ A | 0.04 | 6.28 |  |  |  |  |  |  |  |  |  |  | No |
| **15,594** | R59167 | Meis1, myeloid ecotropic viral integration site 1 homolog 2 (mouse) | MEIS2 | 0.01 | 10.49 |  |  |  |  |  |  |  |  |  | Yes | Yes |
| **15,621** | AA401693 | CD163 molecule | CD163* | 0.02 | 2.46 |  |  |  |  |  |  |  |  |  | Yes | Yes |
| **15,775** | AA485745 | RNA-binding protein | FLJ20273 | 0.04 | -3.81 |  |  |  |  |  |  |  |  |  | Yes | Yes |
| **15,844** | N45218 | Transcribed locus | N/ A | 0.04 | -3.65 |  |  |  |  |  |  |  |  |  |  | No |
| **15,848** | AA496930 | Reversion-inducing-cysteine-rich protein with kazal motifs | RECK | 0.04 | -5.51 | 0.01 | 7.92 | 0.05 | -7.65 | -2.48 | -4.66 | -4.22 | -2.34 |  | Yes | Yes |
| **15,887** | R53428 | ATP-binding cassette, sub-family A (ABC1), member 5 | ABCA5 | 0.03 | 2.54 |  |  |  |  |  |  |  |  |  |  | No |
| **15,899** | W85709 | Solute carrier family 20 (phosphate transporter), member 2 | SLC20A2 | 0.02 | -7.45 |  |  |  |  |  |  |  |  |  |  | No |
| **15,958** | AA425442 | Integrator complex subunit 8 | INTS8 | 0.02 | 7.65 |  |  |  |  |  |  |  |  |  |  | No |
| **16,167** | AA885642 | Histone 1, H2bd | HIST1H2BD | 0 | 2.28 |  |  |  |  |  |  |  |  |  |  | No |
| **16,459** | AA486277 | Retinoblastoma binding protein 5 | RBBP5 | 0.01 | -6.44 | 0 | -12.89 |  |  |  |  |  |  |  |  | No |
| **16,505** | H77818 | Enolase superfamily member 1 | ENOSF1 | 0.01 | 4.49 |  |  |  |  |  |  |  |  |  |  | No |
| **16,531** | H16733 | Transcribed locus | N/ A | 0.04 | -3.73 |  |  |  |  |  |  |  |  |  |  | No |
| **16,547** | AA431887 | BTB (POZ) domain containing 6 | BTBD6 | 0.01 | 7.72 |  |  | 0.05 | 7.41 | -5.02 | -2.86 | -2.57 | -3.04 |  |  | Yes |
| **16,585** | AA701483 | Syntaxin 8 | STX8 | 0.05 | -4.75 |  |  |  |  |  |  |  |  |  |  | No |
| **16,636** | N47013 | Zinc finger protein 569 | ZNF569 | 0.03 | 2.09 |  |  |  |  |  |  |  |  |  |  | No |
| **16,668** | R48312 | Transcribed locus | N/ A | 0.04 | 5.51 |  |  | 0 | 14.8 | -2.04 | -4.96 | -4.39 | -3.12 |  |  | Yes |
| **16,684** | H86812 | Heparan sulfate (glucosamine) 3-O-sulfotransferase 1 | HS3ST1 | 0 | 4.23 |  |  |  |  |  |  |  |  | YES | Yes | Yes |
| **16,711** | AA458853 | RNA binding motif protein 17 | RBM17 | 0.03 | 5.79 | 0.02 | 2.97 |  |  |  |  |  |  |  |  | No |
| **16,713** | R27572 | Alpha-kinase 2 | ALPK2 | 0.04 | 5.43 |  |  |  |  |  |  |  |  |  |  | No |
| **16,813** | R01899 | PAX interacting (with transcription-activation domain) protein 1 | PAXIP1 | 0.04 | -4.12 |  |  |  |  |  |  |  |  |  |  | No |
| **16,833** | N53491 | Sirtuin (silent mating type information regulation 2 homolog) 3 (S. cerevisiae) | SIRT3 | 0.04 | 4.75 |  |  |  |  |  |  |  |  |  |  | No |
| **16,906** | AA461071 | Solute carrier family 23 (nucleobase transporters), member 2 | SLC23A2 | 0.03 | -2.06 | 0 | -2.34 | 0 | -2.09 | 3.68 | 2.58 | 2.61 | 1.34 |  | Yes | Yes |
| **16,925** | R91577 | Chromosome 19 open reading frame 42 | C19orf42 | 0.02 | -4.68 |  |  | 0.04 | -4.15 | -2.77 | -3.73 | -3.89 | -4.05 |  |  | Yes |
| **16,975** | AA485455 | Chromosome 14 open reading frame 118 | C14orf118 | 0.01 | -5.84 |  |  |  |  |  |  |  |  |  |  | No |
| **17,087** | H17785 | Chromosome 14 open reading frame 121 | C14orf121 | 0.03 | -5.96 |  |  |  |  |  |  |  |  |  |  | No |
| **17,158** | N50563 | Adult retina protein | LOC153222 | 0.05 | -3.07 |  |  |  |  |  |  |  |  |  |  | No |
| **17,218** | H92977 | Uracil-DNA glycosylase | UNG | 0.04 | -5.19 |  |  |  |  |  |  |  |  |  |  | No |
| **17,291** | AA704537 | Proteasome (prosome, macropain) subunit, alpha type, 1 | PSMA1 | 0.01 | -6.39 |  |  |  |  |  |  |  |  | YES |  | Yes |
| **17,326** | AA779153 | Homo sapiens, clone IMAGE:5259414, mRNA, mRNA sequence | N/ A | 0.04 | 4.41 |  |  |  |  |  |  |  |  |  |  | No |
| **17,522** | R92011 | Ubiquitin specific peptidase 15 | USP15 | 0.03 | 4.28 |  |  |  |  |  |  |  |  |  |  | No |
| **17,525** | N50152 | OTU domain containing 1 | OTUD1 | 0 | 2.02 | 0.01 | 1.86 |  |  |  |  |  |  |  |  | No |
| **17,665** | H93543 | Brain protein 44-like | BRP44L | 0.01 | 5.75 |  |  |  |  |  |  |  |  |  |  | No |
| **17,676** | H17630 | Piccolo (presynaptic cytomatrix protein) | PCLO | 0.03 | -3.07 |  |  |  |  |  |  |  |  |  |  | No |
| **17,778** | R47938 | Isoprenylcysteine carboxyl methyltransferase | ICMT | 0.05 | -4.16 |  |  | 0.02 | -5.75 | -3.66 | -4.85 | -4.57 | -4.83 |  |  | Yes |
| **17,785** | AA701353 | Hypothetical protein LOC92270 | LOC92270 | 0.02 | 4.54 |  |  |  |  |  |  |  |  |  |  | No |
| **17,803** | R64372 | Membrane-spanning 4-domains, subfamily A, member 7 | MS4A7 | 0 | 5.44 |  |  |  |  |  |  |  |  |  |  | No |
| **17,817** | AA453759 | Sprouty homolog 2 (Drosophila) | SPRY2 | 0.01 | -5.1 |  |  |  |  |  |  |  |  |  |  | No |
| **17,828** | R53027 | Chromosome 1 open reading frame 144 | C1orf144 | 0.01 | -4.34 |  |  |  |  |  |  |  |  |  |  | No |
| **17,838** | R53101 | Chromosome 1 open reading frame 183 | C1orf183 | 0.02 | 4.89 |  |  |  |  |  |  |  |  |  |  | No |
| **17,882** | AI025015 | Transcribed locus | N/ A | 0.03 | 2.3 |  |  |  |  |  |  |  |  |  |  | No |
| **17,887** | AA706094 | Hypothetical protein FLJ25037 | FLJ25037 | 0.01 | 5.8 |  |  |  |  |  |  |  |  |  |  | No |
| **17,994** | W42723 | Chemokine (C-X-C motif) ligand 1 (melanoma growth stimulating activity, alpha) | CXCL1* | 0.02 | 4.64 | 0 | 3.15 | 0 | 17.13 | 1.73 | 5.5 | 3.85 | 7.24 |  |  | Yes |
| **17,997** | AA044052 | Transcribed locus | N/ A | 0.01 | -2.78 | 0 | -1.85 |  |  |  |  |  |  |  |  | No |
| **18,042** | AA598659 | Nuclear mitotic apparatus protein 1 | NUMA1 | 0.04 | 3.58 |  |  |  |  |  |  |  |  | YES | Yes | Yes |
| **18,061** | AA457705 | Immediate early response 3 | IER3 | 0.02 | 2.23 | 0.01 | 1.96 | 0 | 6.48 | 6.25 | 8.81 | 7.34 | 9.76 | YES | Yes | Yes |
| **18,317** | H23464 | UDP-Gal:betaGlcNAc beta 1,3-galactosyltransferase, polypeptide 1 | B3GALT1 | 0.02 | -3.09 |  |  |  |  |  |  |  |  |  |  | No |
| **18,375** | N47445 | Secreted frizzled-related protein 4 | SFRP4 | 0.03 | -2.59 |  |  |  |  |  |  |  |  |  |  | No |
| **18,430** | N62629 | Zinc finger protein 228 | ZNF228 | 0.03 | 3.66 |  |  |  |  |  |  |  |  |  |  | No |
| **18,450** | N59808 | CDNA clone IMAGE:5259272 | N/ A | 0.04 | 3.42 |  |  |  |  |  |  |  |  |  |  | No |
| **18,460** | N62716 | N/A | N/ A | 0.01 | -2.35 |  |  | 0.05 | -2.37 | -2.04 | -4.96 | -4.39 | -3.12 |  |  | Yes |
| **18,470** | N59816 | Peptidase inhibitor 16 | PI16 | 0.01 | -3.06 |  |  |  |  |  |  |  |  |  |  | No |
| **18,531** | AA707167 | Early B-cell factor | EBF | 0.01 | 4.29 | 0 | 3.63 |  |  |  |  |  |  |  |  | No |
| **18,567** | AA923552 | B cell RAG associated protein | GALNAC4S-6ST | 0 | 2.16 | 0 | 2.14 |  |  |  |  |  |  |  | Yes | Yes |
| **18,571** | AA707317 | Phosphodiesterase 4D, cAMP-specific (phosphodiesterase E3 dunce homolog, Drosophila) | PDE4D | 0.02 | 3.56 |  |  | 0.05 | 2.46 | -4.59 | -3.87 | -3.57 | -4.39 |  |  | Yes |
| **18,594** | N62328 | Glutamate receptor, metabotropic 3 | GRM3 | 0 | -2.08 |  |  |  |  |  |  |  |  |  | Yes | Yes |
| **18,643** | R35245 | Alport syndrome, mental retardation, midface hypoplasia and elliptocytosis chromosomal region, gene 1 | AMMECR1 | 0.05 | 2.04 |  |  |  |  |  |  |  |  |  |  | No |
| **18,843** | AA400247 | Chromosome 6 open reading frame 107 | C6orf107 | 0.02 | 2.12 |  |  |  |  |  |  |  |  |  |  | No |
| **18,848** | N29801 | Transcribed locus, strongly similar to NP_068369.1 a disintegrin and metalloproteinase domain 22 isoform 1 preproprotein; metalloproteinase-like, disintegrin-like, and cysteine-rich protein 2 [Homo sapiens] | N/ A | 0 | -3.56 |  |  |  |  |  |  |  |  |  |  | No |
| **18,852** | H20826 | GDP-mannose pyrophosphorylase B | GMPPB | 0 | -2.06 | 0.01 | -1.49 |  |  |  |  |  |  |  | Yes | Yes |
| **18,993** | R65993 | Pregnancy specific beta-1-glycoprotein 9 | PSG9 | 0.03 | -2.31 |  |  | 0.03 | -2.43 | -1.56 | -2.68 | -2.57 | -2.33 |  |  | Yes |
| **19,025** | AA703159 | WD repeat, sterile alpha motif and U-box domain containing 1 | WDSUB1 | 0.05 | 2.18 |  |  |  |  |  |  |  |  |  |  | No |
| **19,064** | N36136 | Endomucin | EMCN | 0.02 | 3.28 |  |  |  |  |  |  |  |  |  |  | No |
