# Supplementary material for: Constitutive Gene Expression in Monocytes from Chronic HIV-1 Infection Overlaps with Acute Toll-Like Receptor Induced Monocyte Activation Profiles
Source: PLoS One. 2012 Jul 18;7(7):e41153. doi: 10.1371/journal.pone.0041153 (PMC3399809; doi:10.1371/journal.pone.0041153)
Supplement: Table S2 — Summary of fold changes in the 82 genes between average expression values for 4 different groups of samples. r = Pearson correlation between expression of a gene and the assigned expression profile (a-f) as “ID” from grouping shown in Figure 3C and also listed on Figure 2. pval = minimal p-value for the gene. (DOC) [file pone.0041153.s002.doc]

**Table SII**

| **Table SII. Summary of fold changes in the 82 genes between average expression values for 4 different groups of samples** | | | | | | | | | |
| --- | --- | --- | --- | --- | --- | --- | --- | --- | --- |
| **ID** | **Accession** | **Symbol** | **Name** | **CS/C** | **PS/P** | **P/C** | **PS/CS** | **pval** | ***r*** |
| a.01 | AA449336 | PRC1 | Protein regulator of cytokinesis 1 | 7.2 | 1.1 | 6.9 | 1.0 | 0.013 | 0.99 |
| a.02 | AA843718 | GTF3C1 | General transcription factor IIIC, polypeptide 1, alpha 220kDa | 3.8 | 1.0 | 4.0 | 1.1 | 0.022 | 0.98 |
| a.03 | H90287 | KDM6A | Lysine (K)-specific demethylase 6A | 5.9 | -1.9 | 7.7 | -1.4 | 0.028 | 0.95 |
| a.04 | AA625655 | REG1A | Regenerating islet-derived 1 alpha | 6.8 | -2.0 | 5.9 | -2.4 | 0.037 | 0.94 |
| a.05 | AA132065 | AA132065 | Transcribed locus | 3.9 | -1.4 | 4.6 | -1.2 | 0.011 | 0.94 |
| a.06 | R83837 | LYN | v-yes-1 Yamaguchi sarcoma viral related oncogene homolog | 2.6 | 1.4 | 2.1 | 1.1 | 0.002 | 0.92 |
| a.07 | AA449440 | IFNGR2 | Interferon gamma receptor 2 (interferon gamma transducer 1) | 3.0 | 1.4 | 2.0 | -1.0 | 4.2x10^-6^ | 0.86 |
| a.08 | AA676604 | MORF4L2 | Mortality factor 4 like 2 | 13.4 | -1.7 | 14.3 | -1.6 | 0.002 | 0.86 |
| a.09 | R48312 | R48312 | Transcribed locus | 14.8 | 1.1 | 5.5 | -2.4 | 0.001 | 0.81 |
| a.10 | AA425102 | CCL2 | Chemokine (C-C motif) ligand 2 | 2.2 | 1.0 | 11.2 | 5.1 | 3.6x10^-6^ | 0.59 |
| b.01 | R38645 | CXorf36 | Chromosome X open reading frame 36 | -4.6 | -1.0 | -4.6 | 1.0 | 0.023 | 1.00 |
| b.02 | AI640779 | SLC12A3 | Solute carrier family 12 (sodium/chloride transporters), member 3 | -3.3 | -1.0 | -3.1 | 1.0 | 0.013 | 1.00 |
| b.03 | R39364 | DCLK1 | Doublecortin-like kinase 1 | -4.9 | -2.0 | -4.1 | -1.7 | 0.035 | 0.99 |
| b.04 | H99490 | MDGA1 | MAM domain containing glycosylphosphatidylinositol anchor 1 | -7.5 | -1.4 | -5.3 | 1.0 | 0.017 | 0.99 |
| b.05 | AA035455 | AA035455 | Transcribed locus | -5.4 | 1.4 | -6.2 | 1.3 | 0.01 | 0.99 |
| b.06 | AA702114 | ERAP1 | Endoplasmic reticulum aminopeptidase 1 | -3.8 | 1.3 | -5.0 | -1.0 | 0.017 | 0.99 |
| b.07 | W86282 | RND3 | Rho family GTPase 3 | -6.6 | -1.2 | -7.0 | -1.3 | 0.026 | 0.99 |
| b.08 | AA682407 | AA682407 | Transcribed locus | -3.8 | 1.8 | -5.9 | 1.1 | 0.023 | 0.98 |
| b.09 | AA406059 | AA406059 | NaN | -4.2 | -2.3 | -7.8 | -4.2 | 0.028 | 0.98 |
| b.10 | R65993 | PSG9 | pregnancy specific beta-1-glycoprotein 9 | -2.4 | 1.2 | -2.3 | 1.3 | 0.027 | 0.98 |
| b.11 | AA401477 | AA401477 | Transcribed locus, strongly similar to NP_065995.1 bile acid beta-glucosidase | -5.1 | -1.2 | -3.8 | 1.1 | 0.026 | 0.97 |
| b.12 | R47938 | RNF207 | Ring finger protein 207 | -5.7 | -1.4 | -4.2 | 1.0 | 0.023 | 0.97 |
| b.13 | N29376 | MNDA | Myeloid cell nuclear differentiation antigen | -3.0 | -2.5 | -2.5 | -2.1 | 7.0x10^-5^ | 0.96 |
| b.14 | AI659114 | TTLL1 | Tubulin tyrosine ligase-like family, member 1 | -7.8 | 2.1 | -3.9 | 4.3 | 0.028 | 0.94 |
| b.15 | N54165 | DEFA1 | Defensin, alpha 1 | -17.9 | -3.5 | -5.8 | -1.1 | 0.001 | 0.94 |
| b.16 | AI084074 | BLK | B lymphoid tyrosine kinase | -3.6 | 1.9 | -3.6 | 1.9 | 0.036 | 0.94 |
| b.17 | R52542 | IMPDH1 | IMP (inosine monophosphate) dehydrogenase 1 | -2.2 | 2.1 | -3.2 | 1.4 | 0.006 | 0.94 |
| b.18 | AA461071 | SLC23A2 | Solute carrier family 23 (nucleobase transporters), member 2 | -2.1 | -2.4 | -2.1 | -2.3 | 1.3x10^-5^ | 0.92 |
| b.19 | N47443 | ZNF536 | Zinc finger protein 536 | -6.8 | 3.0 | -8.2 | 2.5 | 0.009 | 0.92 |
| b.20 | H89996 | H89996 | Transcribed locus | -7.6 | 2.5 | -5.2 | 3.6 | 0.014 | 0.91 |
| b.21 | N62716 | N62716 | NaN | -2.4 | -1.5 | -2.3 | -1.5 | 0.01 | 0.90 |
| b.22 | AA973337 | PCF11 | PCF11, cleavage and polyadenylation factor subunit, homolog (S. cerevisiae) | -3.9 | 1.5 | -9.7 | -1.7 | 0.002 | 0.90 |
| b.23 | AI017154 | NTSR2 | Neurotensin receptor 2 | -9.6 | 1.5 | -6.2 | 2.3 | 0.007 | 0.90 |
| b.24 | N71796 | FCRLA | Fc receptor-like A | -12.2 | 2.1 | -7.9 | 3.2 | 0.009 | 0.90 |
| b.25 | AA428196 | POU4F1 | POU class 4 homeobox 1 | -4.4 | 2.0 | -3.2 | 2.8 | 0.022 | 0.83 |
| b.26 | N75498 | C16orf45 | Chromosome 16 open reading frame 45 | -4.6 | 3.7 | -11.8 | 1.4 | 0.003 | 0.71 |
| b.27 | R91577 | C19orf42 | Chromosome 19 open reading frame 42 | -4.2 | -1.0 | -4.7 | -1.2 | 0.023 | 0.51 |
| c.01 | AA063573 | SAMSN1 | SAM domain, SH3 domain and nuclear localization signals 1 | 2.1 | 1.8 | 2.4 | 2.0 | 0.005 | 1.00 |
| c.02 | AI815076 | SLC7A7 | Solute carrier family 7 (cationic amino acid transporter, y+ system), member 7 | 2.0 | 1.8 | 2.3 | 2.1 | 0.004 | 1.00 |
| c.03 | AA683578 | ADA | Adenosine deaminase | 3.5 | 2.9 | 4.2 | 3.4 | 3.9x10^-6^ | 1.00 |
| c.04 | AA935273 | CXCL3 | Chemokine (C-X-C motif) ligand 3 | 9.8 | 7.9 | 3.7 | 3.0 | 4.1x10^-6^ | 0.99 |
| c.05 | AA489629 | NAMPT | Nicotinamide phosphoribosyltransferase | 3.2 | 2.9 | 2.3 | 2.1 | 0.001 | 0.99 |
| c.06 | AI285199 | CCL20 | Chemokine (C-C motif) ligand 20 | 74.8 | 33.1 | 10.2 | 4.5 | 4.3x10^-8^ | 0.99 |
| c.07 | W46900 | CXCL1 | Chemokine (C-X-C motif) ligand 1 | 15.4 | 8.3 | 5.2 | 2.8 | 2.2x10^-6^ | 0.99 |
| c.08 | W95041 | HS3ST3B1 | Heparan sulfate (glucosamine) 3-O-sulfotransferase 3B1 | 5.5 | 6.4 | 3.0 | 3.5 | 7.2x10^-6^ | 0.98 |
| c.09 | AA398218 | NME3 | Non-metastatic cells 3, protein expressed in | 7.8 | 9.6 | 3.2 | 3.9 | 2.0x10^-7^ | 0.98 |
| c.10 | W46900 | CXCL1 | Chemokine (C-X-C motif) ligand 1 | 13.7 | 10.5 | 4.4 | 3.4 | 3.1x10^-7^ | 0.98 |
| c.11 | AA935273 | CXCL3 | Chemokine (C-X-C motif) ligand 3 | 11.6 | 8.6 | 3.7 | 2.7 | 1.1x10^-6^ | 0.98 |
| c.12 | N49405 | NEDD8 | Neural precursor cell expressed, developmentally down-regulated 8 | 25.3 | 8.3 | 9.9 | 3.3 | 1.6x10^-6^ | 0.98 |
| c.13 | N73680 | SLC11A2 | Solute carrier family 11, member 2 | 2.5 | 2.3 | 2.0 | 1.8 | 0.0002 | 0.98 |
| c.14 | H59780 | HS3ST3B1 | Heparan sulfate (glucosamine) 3-O-sulfotransferase 3B1 | 6.2 | 5.9 | 2.8 | 2.7 | 1.1x10^-7^ | 0.98 |
| c.15 | AA425900 | CCNO | Cyclin O | 10.6 | 9.5 | 3.7 | 3.3 | 2.1x10^-8^ | 0.98 |
| c.16 | AA453293 | PDE4B | Phosphodiesterase 4B, cAMP-specific | 4.3 | 4.1 | 2.9 | 2.7 | 8.6x10^-7^ | 0.98 |
| c.17 | AA973928 | IGHMBP2 | Immunoglobulin mu binding protein 2 | 5.8 | 8.2 | 2.2 | 3.2 | 1.1x10^-5^ | 0.97 |
| c.18 | AA410375 | GMPR | Guanosine monophosphate reductase | 3.1 | 6.4 | 3.1 | 6.2 | 4.5x10^-6^ | 0.97 |
| c.19 | AA018591 | SPTBN1 | Spectrin, beta, non-erythrocytic 1 | 17.2 | 10.3 | 4.8 | 2.9 | 3.0x10^-6^ | 0.97 |
| c.20 | AA404239 | ENAH | Enabled homolog (Drosophila) | 8.6 | 8.8 | 3.3 | 3.4 | 8.9x10^-8^ | 0.97 |
| c.21 | T49159 | SERPINB2 | Serpin peptidase inhibitor, clade B (ovalbumin), member 2 | 3.1 | 5.7 | 4.0 | 7.5 | 3.0x10^-6^ | 0.96 |
| c.22 | AI889554 | CXCL6 | Chemokine (C-X-C motif) ligand 6 (granulocyte chemotactic protein 2) | 4.8 | 2.1 | 6.5 | 2.9 | 0.004 | 0.96 |
| c.23 | N98591 | IL6 | interleukin 6 (interferon, beta 2) | 18.7 | 75.7 | 3.2 | 13.1 | 1.7x10^-7^ | 0.95 |
| c.24 | W42723 | CXCL1 | Chemokine (C-X-C motif) ligand 1 | 17.1 | 11.6 | 4.6 | 3.1 | 3.2x10^-6^ | 0.95 |
| c.25 | H62864 | CCL4L1 | Chemokine (C-C motif) ligand 4-like 1 | 29.7 | 31.7 | 2.3 | 2.5 | 4.9x10^-9^ | 0.93 |
| c.26 | H62864 | CCL4L1 | Chemokine (C-C motif) ligand 4-like 1 | 33.8 | 27.1 | 2.9 | 2.3 | 9.8x10^-9^ | 0.92 |
| c.27 | AI992097 | CYP11A1 | cytochrome P450, family 11, subfamily A, polypeptide 1 | 5.3 | 1.3 | 4.5 | 1.1 | 0.029 | 0.92 |
| c.28 | AA457705 | IER3 | Immediate early response 3 | 6.5 | 5.7 | 2.2 | 2.0 | 8.4x10^-7^ | 0.91 |
| c.29 | W46900 | CXCL1 | Chemokine (C-X-C motif) ligand 1 | 28.2 | 4.7 | 8.9 | 1.5 | 8.4x10^-6^ | 0.90 |
| c.30 | AA489068 | N-PAC | Cytokine-like nuclear factor n-pac | 2.4 | 1.7 | 4.6 | 3.4 | 0.001 | 0.87 |
| c.31 | AA521384 | SLC39A8 | Solute carrier family 39 (zinc transporter), member 8 | 11.0 | 2.8 | 4.9 | 1.3 | 6.5x10^-6^ | 0.78 |
| d.01 | AA609992 | DHRS9 | Dehydrogenase/reductase (SDR family) member 9 | -2.0 | -2.4 | -3.6 | -4.1 | 0.001 | 0.97 |
| e.01 | W93369 | DKFZP434L187 | Hypothetical LOC26082 | 7.3 | -3.9 | 7.5 | -3.8 | 0.016 | 0.97 |
| e.02 | AA707317 | PDE4D | phosphodiesterase 4D, cAMP-specific | 2.5 | -2.6 | 3.6 | -1.8 | 0.015 | 0.96 |
| e.03 | N29914 | EDNRB | Endothelin receptor type B | 11.6 | -2.0 | 6.6 | -3.6 | 0.008 | 0.94 |
| e.04 | AA521243 | MRPL19 | Mitochondrial ribosomal protein L19 | 9.5 | -2.3 | 8.7 | -2.5 | 0.005 | 0.94 |
| e.05 | AA431887 | BTBD6 | BTB (POZ) domain containing 6 | 7.4 | -1.3 | 7.7 | -1.3 | 0.015 | 0.91 |
| e.06 | N95761 | FUCA1 | Fucosidase, alpha-L- 1, tissue | -2.2 | -6.0 | 4.5 | 1.7 | 5.3x10^-6^ | 0.51 |
| f.01 | AI659563 | AR | Androgen receptor | -2.7 | 3.0 | -3.0 | 2.7 | 0.02 | 1.00 |
| f.02 | AA496930 | RECK | Reversion-inducing-cysteine-rich protein with kazal motifs | -7.6 | 5.7 | -5.5 | 7.9 | 0.006 | 0.97 |
| f.03 | AA972350 | SFTPB | Surfactant protein B | -5.6 | 2.7 | -5.4 | 2.8 | 0.02 | 0.96 |
| f.04 | N36083 | GTDC1 | glycosyltransferase-like domain containing 1 | -11.1 | 2.9 | -6.3 | 5.0 | 0.016 | 0.95 |
| f.05 | N34415 | FRMD4B | FERM domain containing 4B | -15.9 | 3.1 | -7.8 | 6.3 | 0.004 | 0.95 |
| f.06 | AA521026 | OGG1 | 8-oxoguanine DNA glycosylase | -3.6 | 4.1 | -7.3 | 2.0 | 0.003 | 0.88 |
| f.07 | AA131885 | C2orf86 | Chromosome 2 open reading frame 86 | -5.2 | 6.2 | -7.6 | 4.3 | 0.01 | 0.79 |
